# Functional Nanomaterials for Advanced Bioelectrode Interfaces: Recent Advances in Disease Detection and Metabolic Monitoring

**DOI:** 10.3390/s25144412

**Published:** 2025-07-15

**Authors:** Junlong Ma, Siyi Yang, Zhihao Yang, Ziliang He, Zhanhong Du

**Affiliations:** 1 The Brain Cognition and Brain Disease Institute (BCBDI), Shenzhen Institutes of Advanced Technology, Chinese Academy of Sciences, Shenzhen 518055, China; majlong@mail2.sysu.edu.cn (J.M.); sy.yang2@siat.ac.cn (S.Y.); leo_yang2020@163.com (Z.Y.); 12433439@mail.sustech.edu.cn (Z.H.); 2 Guangdong Provincial Key Laboratory of Brain Connectome and Behavior, Shenzhen Institute of Advanced Technology, Chinese Academy of Sciences, Shenzhen 518055, China; 3 CAS Key Laboratory of Brain Connectome and Manipulation, Shenzhen Institute of Advanced Technology, Chinese Academy of Sciences, Shenzhen 518055, China; 4 Shenzhen-Hong Kong Institute of Brain Science, Shenzhen 518055, China; 5 Shenzhen Fundamental Research Institutions, Shenzhen 518055, China; 6 School of Life Sciences, Southern University of Science and Technology, Shenzhen 518055, China

**Keywords:** nanomaterials, bioelectrodes, electrophysiology, electrochemistry, multimodality, diagnostics, monitoring, wearables

## Abstract

As critical interfaces bridging biological systems and electronic devices, the performance of bioelectrodes directly determines the sensitivity, selectivity, and reliability of biosensors. Recent advancements in functional nanomaterials (e.g., carbon nanomaterials, metallic nanoparticles, 2D materials) have substantially enhanced the application potential of bioelectrodes in disease detection, metabolic monitoring, and early diagnosis through strategic material selection, structural engineering, interface modification, and antifouling treatment. This review systematically examines the latest progress in nanomaterial-enabled interface design of bioelectrodes, with particular emphasis on performance enhancements in electrophysiological/electrochemical signal acquisition and multimodal sensing technologies. We comprehensively analyze cutting-edge developments in dynamic metabolic parameter monitoring for chronic disease management, as well as emerging research on flexible, high-sensitivity electrode interfaces for early disease diagnosis. Furthermore, this work focused on persistent technical challenges regarding nanomaterial biocompatibility and long-term operational stability while providing forward-looking perspectives on their translational applications in wearable medical devices and personalized health management systems. The proposed framework offers actionable guidance for researchers in this interdisciplinary field.

## 1. Introduction

Biosensors serve as essential analytical tools extensively employed across life sciences, medical diagnostics, and health-monitoring domains. Electrophysiological sensors enable real-time capture of bioelectrical signals, providing dynamic monitoring capabilities for pathological states and physiological processes. Recent advancements in materials science and microelectronics have driven a paradigm shift in biosensor design—transitioning from conventional macroscale materials toward flexible, high-efficiency nanomaterials and microstructures [1,2] that help to enhance sensor performance metrics and operational adaptability. Bioelectrical signals—including action potentials in neurons/muscles, field potentials from cardiac tissue, and metabolism-associated ionic currents—provide critical insights into cellular activities and organ functionality [3]. These signals constitute direct manifestations of fundamental biochemical processes within organisms, thereby playing pivotal roles in monitoring neurological disorders. Their detection and interpretation serve as powerful clinical diagnostic tools, enabling early warnings and continuous monitoring for conditions such as epilepsy, cardiac arrest, and Alzheimer’s disease.

Biosensors facilitate precise monitoring of biomarkers including glucose [4], neurotransmitters [5,6,7], and ions [3], supporting disease management. Their high-precision, high-sensitivity in situ detection capabilities empower medical professionals to sensitively perceive disease onset and progression, substantially aiding early diagnosis. The development of long-lifetime devices holds significant value for chronic disease monitoring and continuous health assessment, thereby advancing personalized healthcare.

Conventional biosensors primarily rely on materials such as metals (e.g., platinum, gold) or inorganic compounds. While these materials exhibit favorable electrical conductivity and chemical stability, they generally suffer from inherent limitations, including low biocompatibility, poor long-term stability, high mechanical rigidity, and elevated interfacial impedance [8]. Although metal electrodes exhibit excellent electrical conductivity, their poor biocompatibility often leads to inflammatory responses and encapsulation by scar tissue, resulting in reduced surface activity of the electrode. Furthermore, the mechanical mismatch between rigid metals and soft tissues can cause increased interfacial impedance, impairing signal transmission. For example, platinum electrodes commonly used in deep brain stimulation (DBS) applications may experience surface passivation during long-term implantation, which compromises signal conduction [8]. To address the challenges of biocompatibility and mechanical rigidity, growing research efforts have focused on exploring flexible, biodegradable materials to enhance the biocompatibility and long-term stability of electrodes. Flexible materials such as conductive polymers [9] and hydrogels [10] have been increasingly employed in the fabrication of biosensors. However, compared to metal electrodes, their electrical conductivity is significantly reduced, making it challenging to directly fabricate microelectrodes with adequate performance. Moreover, while certain conductive polymers (e.g., poly(3,4-ethylenedioxythiophene)-poly(styrenesulfonate) (PEDOT:PSS)) can mitigate impedance issues, they often exhibit insufficient long-term stability.

In recent years, nanomaterials, due to their unique physicochemical properties and precise structural design, have addressed some limitations of macroscopic materials and achieved notable performance improvements [11], thereby demonstrating substantial application potential and practical utility.

Nanomaterials represent a class of materials characterized by exhibiting at least one dimension within the nanoscale range (1–100 nm). Common examples include carbon nanotubes, graphene, MXenes, and metal nanoparticles, which possess unique geometric architectures. Concurrently, these materials demonstrate distinctive properties across multiple domains, such as electrical conductivity, specific surface area, biocompatibility [12], and catalytic activity [13], rendering them highly promising for applications in bioelectronic sensors and attracting substantial research interest from the scientific community. Graphene’s excellent charge transport and biocompatibility enable high-fidelity neural signal detection and pathological monitoring [14,15]. MXene-based electrodes achieve enhanced charge storage capacity and conductivity, significantly reducing impedance while improving signal acquisition [16]. Metal nanoparticles boost sensitivity through catalytic amplification, elevating sensor performance [17]. Beyond the intrinsic properties of materials, the unique structural morphologies of nanomaterials enable the performance enhancement of biosensors through structural design strategies, including increasing active surface area [18] and constructing porous anti-biofouling features [19]. However, nanomaterials also present several inherent drawbacks. For example, while carbon-based nanomaterials can enhance bioelectrode interfaces, insufficient surface chemical modification may induce nonspecific protein adsorption, leading to biofouling issues that compromise electrode performance [20]. MXene materials, conversely, exhibit lower stability and proneness to oxidation [21], which hinders their ability to achieve long-term in vivo recording. Consequently, functionalization modifications of nanomaterials and material composite techniques have emerged as major foci of research. These advancements enable electrodes fabricated from nanomaterials to achieve higher selectivity and longer-term stability [22] in complex biological environments. Such tunable characteristics make them particularly suitable for the fabrication of flexible biosensors, which is critical for wearable and implantable devices and one of the prerequisites for realizing long-term monitoring capabilities.

Building upon advancements in single-signal sensing accuracy and longevity, multimodal sensors integrate diverse functional materials and sensing elements to simultaneously monitor multiple biological signals including electrophysiological activities, chemical biomarkers, and thermal changes [23]. This approach delivers coupled physiological insights essential for comprehensively understanding disease mechanisms and therapeutic efficacy, thereby supporting early diagnosis and real-time intervention for neurological disorders.

Such modular designs can incorporate complementary therapeutic modules utilizing optical, electrical, thermal, or pharmaceutical delivery modalities [23]. When integrated into wearable or implantable platforms, these sensors enable continuous real-time multiparametric health monitoring, demonstrating considerable promise for early disease detection, therapeutic management, and personalized treatment regimens.

Here, we have completed a review of the recent development status of electrode materials and structures, and pointed out the important position of current research topics in the development history of electrophysiological/electrochemical electrodes (Table 1).

This review synthesizes recent advancements in biosensors employing nanomaterials, with particular emphasis on their pivotal roles in enhancing electrophysiological/electrochemical signal acquisition and multimodal sensing capabilities. We first systematically summarize frontier developments in functional nanomaterials for bioelectrode design, analyzing current applications in electrophysiological and electrochemical sensors. Subsequently, we examine performance enhancements and developmental potential derived from consciously engineered microstructures in bioelectrodes. We then explore the integration of nanomaterials into clinically viable multimodal devices, highlighting the capacity for cross-validated high-precision sensing to elevate system reliability. Finally, we spotlight cutting-edge applications in clinical and health management domains while envisioning next-generation biosensors leveraging novel nanomaterials and refined microstructural designs—aiming to advance practical implementations in wearable medical devices and personalized health management systems.

## 2. Progress of Functional Nanomaterials in the Design of Biological Electrodes

### 2.1. Electrophysiological Sensing Nanomaterials

Nanomaterials are primarily utilized for electrode sites and conductive pathways in electrophysiological sensors. The high chemical reactivity [36] and large specific surface area [37] resulting from the size effects of nanomaterials significantly enhance sensor–bio-interface performance, offering advantages such as high sensitivity and stability [38]. Certain conductive nanomaterials can function as conductive layers or serve as conductive reinforcing phases that establish conductive networks within composite materials [39], thereby improving the mechanical and electrical properties of flexible electrodes.

#### 2.1.1. Non-Metallic Nanomaterials

Carbon-based nanomaterials, as one of the most common conductive nanomaterials, have diverse morphological and structural features such as carbon quantum dots, carbon nanotubes [40], and graphene. While maintaining excellent electrical conductivity, these materials possess abundant surface functional groups and favorable biocompatibility [41], making them among the most frequently used materials in electrophysiological sensors [42]. Graphene, as a prominent material in this category, has garnered extensive research and application in the field of electrophysiological electrodes [43,44].

**Figure 1:**
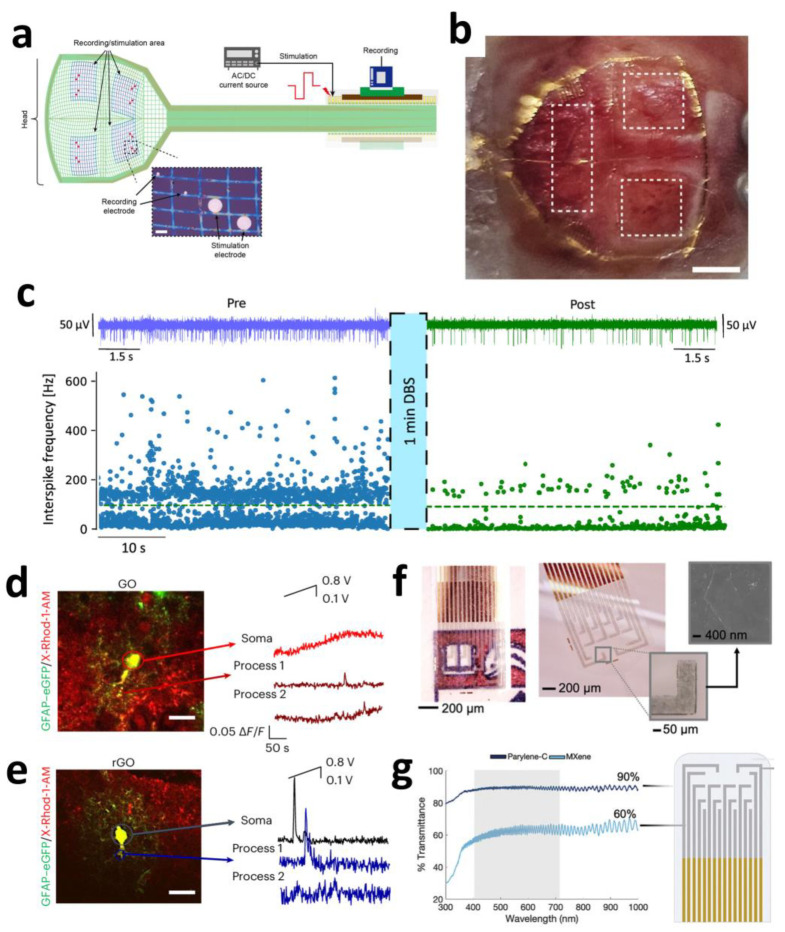
(**a**) Schematic for internal electrical stimulation and recording using LNW. (**b**) Optical image of the LNW and the entire interface on the mouse brain. Scale bar: 1 cm [45]. Copyright © 2025, American Chemical Society. (**c**) Effect of microelectrode stimulation on the spike firing activity, pre (blue trace) and post (green trace) stimulation [46]. Copyright © 2025, the author(s). (**d**,**e**) Representative traces of [Ca^2+^]_i_ over time (**right**) performed with high magnification on X-Rhod-1/GFAP–eGFP-labeled astrocytes (merged images, **left**) for slices on GO (**d**) and on rGO (**e**), analyzed in astrocytic soma and in astrocytic processes [47]. Copyright © 2024, the author(s). (**f**) Digital microscopy images of the complete arrays. Inset is the SEM image of a Ti_3_C_2_T*_x_* flake on the electrode site. (**g**) Optical transmittance spectra of the Ti_3_C_2_T*_x_* electrode contacts and Parylene-C substrate [54]. Copyright © 2024, the author(s).

Carbon-based nanomaterials exhibit exceptionally high application value in cortical electrodes. Large-scale, multi-regional synchronous neural recording with high resolution is crucial for understanding complex neural circuit functions; however, conventional surface electrode arrays (SEAs) suffer from limited spatial resolution and restricted coverage areas. Ultra-thin graphene-based probes offer a novel solution to this challenge. The “Large-area NeuroWeb” (LNW) probe developed by Pyo et al. comprises four independent recording regions, each containing 16 platinum electrodes (totaling 64 channels for the device). These electrodes are interconnected via a graphene (Gr) network and encapsulated between top and bottom insulating layers of hexagonal boron nitride (h-BN) (Figure 1a). With a total thickness of merely ~100 nm and an area of ~9 × 9 mm^2^, the device can cover a substantial portion of the mouse cerebral cortex (Figure 1b).

Attributed to graphene’s properties, the ultra-thin, flexible structure conforms tightly to the brain’s curved surface, significantly reducing tissue displacement and inflammatory responses. The h-BN/Gr/h-BN architecture provides exceptional electrical performance and encapsulation reliability, meeting the demands of high-sensitivity neural signal detection. This integrated design enables simultaneous recording of high-quality unit action potentials from multiple discrete brain regions while maintaining a stable operation for over 7 days. It simplifies experimental procedures and mitigates the risks of additional tissue damage and signal interference associated with multi-probe implants. Through whisker deflection and electrical stimuli experiments, the LNW probe further demonstrated bidirectional neuronal interfacing capabilities (recording and stimulation), showcasing its potential for closed-loop brain–machine interfaces (BMIs) and neural prosthetics [45].

Beyond cortical electrodes, carbon-based nanomaterials exhibit significant potential in deep brain region electrodes. DBS represents an effective therapy for movement disorders such as Parkinson’s disease (PD), yet conventional clinical DBS electrodes (typically Pt- or PtIr-based) suffer from large dimensions (millimeter-scale) and low spatial resolution, hindering precise targeting of small deep nuclei and high-fidelity neural signal monitoring. Ria et al. demonstrated a breakthrough application of flexible high-density microelectrode arrays based on nanoporous reduced graphene oxide (rGO) for recording and stimulation in deep brain regions (specifically the subthalamic nucleus, STN). This electrode array features a flexible polyimide substrate measuring only 120 μm in width and 10 μm in thickness, with eight 25 μm diameter rGO microelectrodes (100 μm spacing) integrated at its tip. The nanoporous structure of rGO provides an extensive electrochemical active surface area, enabling ultralow impedance (~29.4 kΩ @1 kHz in PBS) and high-fidelity recording capabilities (spike signal-to-noise ratio > 10). Furthermore, rGO confers an ultra-high charge injection limit (CIL) of 2.3 mC/cm^2^, substantially surpassing planar metal microelectrodes. Additionally, this rGO array demonstrates robust bidirectional functionality. Beyond capturing Parkinson’s-specific neural biomarkers (e.g., enhanced burst-like activity in the STN), it selectively modulates (suppresses) such activity through high-density focal electrical stimulation (simulating standard high-frequency DBS) (Figure 1c). This localized neuromodulation capability targeting specific calcium-dependent signaling pathways, combined with high-resolution recording, establishes a promising technological platform for biomarker-driven closed-loop adaptive DBS [46].

Beyond recording and stimulating neuronal activity, understanding and modulating calcium signaling in glial cells—particularly astrocytes—is critical for deciphering neuro-glial interactions and developing novel neuromodulation strategies. Carbon-based nanomaterials offer exceptional value in this domain. Research by Fabbri et al. revealed the unique capability of graphene oxide (GO) and reduced graphene oxide (rGO) electrodes to selectively activate distinct astrocyte calcium signaling pathways. This divergence stems from differences in interface electric field distributions governed by the distinct conductivity of GO and rGO. At the insulating GO interface, the electric field concentrates at the electrode–cell boundary, effectively depolarizing the plasma membrane and opening voltage-gated calcium channels. Conversely, the conductive rGO interface distributes the field primarily at the cell–electrolyte interface, potentially triggering endoplasmic reticulum (ER) calcium release directly or indirectly (e.g., via mechano-/osmotic-mediated effects), while inhibiting extracellular calcium influx.

This ability to leverage the intrinsic electrical properties of electrode materials (insulating GO vs. conductive rGO) for pathway-specific activation (Figure 1d,e) provides a precise interrogative tool to investigate the functional roles of astrocytic calcium signals in neurovascular coupling, synaptic plasticity, and neurological disorders [47].

Similar to graphene, MXene is a biocompatible, highly conductive two-dimensional nanomaterial that has garnered increasing research attention in recent years [48]. Compared to carbon-based materials, MXene features abundant hydrophilic surface groups [49], transition metal layers that confer catalytic activity [50], and self-assembled lamellar stacked architectures that form stable flexible conductive networks [51]. Its electromagneticwave absorption capabilities further suggest potential value in photothermal therapy [52]. These properties position MXene as a promising material for electrophysiological/electrochemical sensors and theranostic devices [53].

MXene’s optical transparency under specific conditions enables the fabrication of transparent electrode arrays for multimodal neural monitoring, offering unique optoelectronic synergy. Shankar et al. developed solution-processed transparent Ti_3_C_2_T_x_ MXene microelectrodes (60% transmittance at 550 nm) that simultaneously achieve low impedance (563 kΩ@1 kHz) and high charge storage capacity (58 mC cm^−2^), effectively overcoming optical occlusion and photoelectric artifacts inherent to metal electrodes (Figure 1f,g). In epileptic rat models, these electrodes enabled synchronized electrophysiological recording and calcium imaging, precisely capturing the dynamic propagation of epileptic wavefronts. Owing to MXene’s exceptionally low light absorption in the visible spectrum (400–600 nm) coupled with its high conductivity, it emerges as a transformative tool for synchronized electro-optical studies of neural circuits [54].

Modification techniques targeting MXene itself can impart superior properties, while surface treatments further expand its functional capabilities. Wu et al. proposed an orbital symmetry-breaking strategy that converts MXene into OBXene via oxygen plasma treatment, achieving closed-loop theranostics in a porcine myocardial infarction model. The modified OBXene exhibited a 25% reduction in interfacial impedance (144 Ω) and activated a Schottky interfacial piezoelectric effect, lowering the pressure detection threshold of infused ionogels to 371 Pa. Implantation studies demonstrated that OBXene reduced the expression of inflammatory biomarkers by 40% and decreased fibrotic thickness by 35%. The engineered cardiac patch employed wireless/battery-free operation to monitor myocardial contractile anomalies in real-time, triggering precise electrical stimulation therapy autonomously. This integrated “detection-treatment” system represents a complete implantable device solution for myocardial infarction management [55].

#### 2.1.2. Metallic Nanomaterials

Compared to non-metallic materials such as carbon-based nanomaterials, metallic nanomaterials possess distinct advantages. The properties of metallic nanoparticles (NPs), nanorods (NRs), and nanowires (NWs) vary significantly depending on their morphology [56]. While NPs and NRs are primarily used in electrochemical sensing, NW-based conductive networks exhibit relatively high electrical conductivity [57] and substantial stretchability [58], making them particularly valuable for flexible electrophysiological sensors. Silver nanomaterials, for example, offer inherent antimicrobial properties [59], which help suppress infection-induced inflammation and facilitate long-term wearable medical sensing.

**Figure 2:**
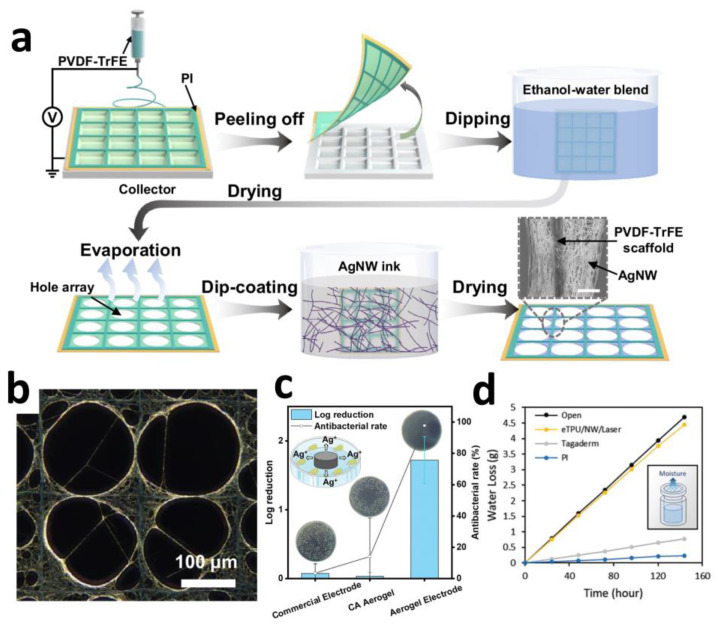
(**a**) Schematic illustration of the fabrication process for AgNW networks, and the inserted SEM image of AgNW wrapped around PVDF-TrFE scaffold. Scale bar: 5 μm. (**b**) SEM images depict the electrical pathways under stretching. Scale bar: 100 μm [60]. Copyright © 2024, the author(s). (**c**) In vitro antibacterial activity studies of aerogel electrode and antibacterial rate of aerogel electrode [61]. Copyright © 2024, Wiley-VCH. (**d**) Breathability comparison of popular substrate materials for epidermal electronics [62].Copyright © 2024, Wiley-VCH.

Highly conductive networks formed by metallic nanowires enable ultra-thin electrode designs that enhance tissue conformability. Qin et al. employed an evaporation-induced self-assembly strategy to construct ordered silver nanowire (AgNW) networks on freestanding electrospun PVDF-TrFE frameworks (Figure 2a). This architecture achieved an ultralow thickness of 5 μm, 87.8% optical transmittance, and sheet resistance as low as 8.4 Ω·sq^−1^. The aligned nanowire network maintained electrical pathway integrity under 50% tensile strain (resistance change < 0.96% after 50,000 cycles) (Figure 2b), while its low bending stiffness (≈1 MPa) ensured conformal adhesion to curved surfaces such as the epicardium, significantly minimizing motion artifacts [60].

Certain metallic nanomaterials exhibit intrinsic antimicrobial properties that suppress inflammatory responses. Wang et al. developed electrodes comprising cellulose aerogel-supported polypyrrole-coated silver nanowires (AgNWs@PPy-CA), demonstrating potent antibacterial characteristics. In vitro tests revealed that the synergistic effect of sustained Ag^+^ release and PPy achieved an antibacterial rate > 97.8% (Figure 2c), significantly exceeding that of commercial electrodes. Murine models confirmed its efficacy in suppressing neutrophil infiltration and reducing the expression of inflammatory cytokines TNF-α and IL-6 [61].

Leveraging the intrinsic high conductivity of metallic materials, conductive networks enable breathable and stretchable structural designs. Yoon et al. developed a nanowire direct local filtering technique for patterning Ag@Au core–shell nanowires on electrospun thermoplastic polyurethane membranes (eTPU). Capillary forces generated by carbon paper drive selective nanowire deposition, while laser embedding enhances interfacial bonding strength. This achieves a water vapor transmission rate of 4015 g·m^−2^·d^−1^ (exceeding skin perspiration by 9-fold) (Figure 2d). Combined with an ECaP electrode layer design, the electrode maintains impedance variation < 5% under 150% strain, resolving the longstanding challenge of concurrently achieving stretchability and electrical stability [62].

**Figure 3:**
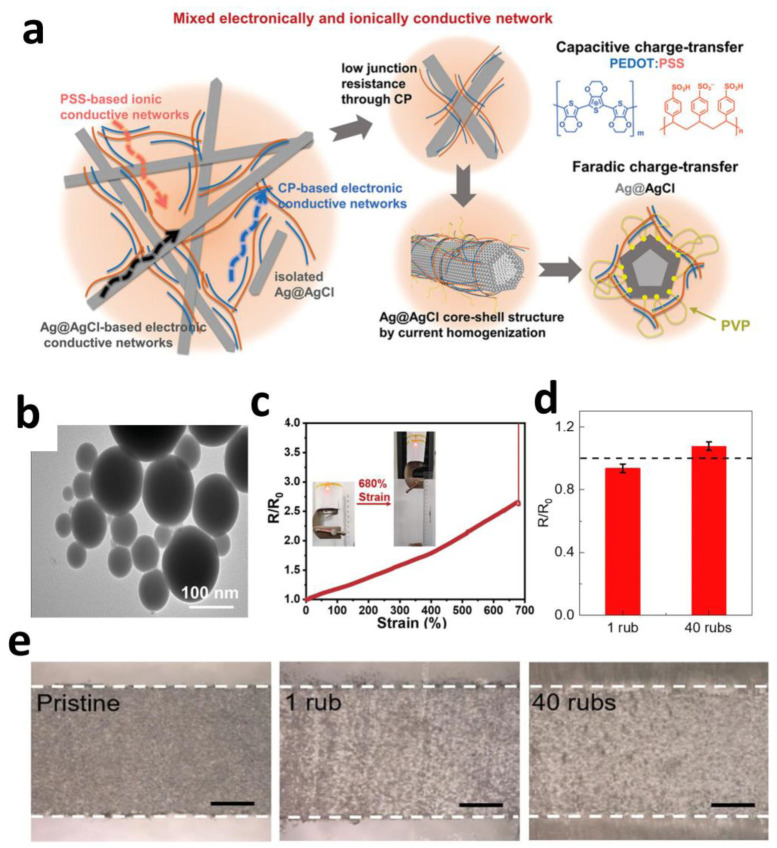
(**a**) Schematic illustration of CP-Ag@AgCl electrode [63]. Copyright © 2024, Wiley-VCH. (**b**) Transmission electron microscope (TEM) image of EGaIn NPs. (**c**) Resistance of the conductive path under different tensile strains [68]. Copyright © 2023, Wiley-VCH. (**d**) Normalized resistance of the nanocomposite with respect to the number of rubs. (**e**) Optical images of the nanocomposite in its original, after 1 rub and after 40 rubs. Scale bars: 500 µm [69]. Copyright © 2024, Elsevier.

Certain modified metallic nanomaterials, when combined with other materials, enable high-fidelity signal transduction mechanisms in electrodes. Hu et al. developed a CP–Ag@AgCl composite electrode (Figure 3a) that achieves broadband frequency response through dual-charge transfer pathways. PEDOT:PSS provides pseudocapacitive behavior in high-frequency regions (>100 Hz) with an areal capacitance of 0.28 F·m^−2^, while Ag@AgCl nanowires maintain stable electrode potential in low-frequency regimes (<1 Hz) with minimal drift (<4.3 mV). This synergistic effect yields a signal-to-noise ratio (SNR) of 26.5 dB (suitable for ECG) and a charge injection capacity (CIC) > 0.32 mC·cm^−2^ (exceeding neuromodulation thresholds). During bidirectional signal processing, post-stimulation electrode potential shifts remain < 5 mV, overcoming signal saturation issues caused by potential drift in conventional electrodes [63].

Metallic nanomaterials exhibit exceptional intrinsic properties that enable multifunctional capabilities through strategic design: flexibility (ultra-thin/stretchable designs), high electrical performance (low impedance/high fidelity), broad frequency response, antibacterial/anti-inflammatory properties, and breathability. These features establish a material foundation for long-term stable, high-precision biosignal monitoring—particularly suitable for wearable health monitoring and human-machine interfaces. Future research should focus on enhancing long-term operational stability, scalable manufacturing processes, and multimodal sensor integration.

Beyond solid metallic nanoparticles, liquid metals form nanostructured particles via ultrasonication [64] or mechanical agitation [65]. Their liquid nature imparts extreme flexibility [66], conformability [67], and self-healing capabilities, making them exceptionally valuable for electrophysiological electrodes and particularly e-skin applications.

Liquid metal nanoparticles confer electrodes with extreme deformability, enabling highly stretchable conductive networks on elastic substrates. Niu et al. developed a “thermal sintering” EGaIn nanoparticle (EGaIn NP) ink (Figure 3b) by incorporating thermal expansion microspheres (TEMs) into the EGaIn NP solution. Heating-induced TEMs expansion generates mechanical pressure that effectively ruptures the NPs’ oxide shells, promoting their fusion into continuous conductive pathways. The resulting bioelectrode arrays exhibited astonishing mechanical and electrical performance, withstanding tensile strains up to 680% (Figure 3c)—far exceeding human joint motion ranges (30–100%)—providing a viable material solution for dynamic physiological signal monitoring [68].

Certain liquid metals demonstrate exceptional cyclic stability and favorable biocompatibility, showing significant potential for bioelectrode applications. Zheng et al. proposed a strategy utilizing semi-embedded liquid metal particles within polymer nanofiber membranes. Recognizing that the confinement morphology of liquid metal nanoparticles within fibrous networks critically determines long-term stability, the researchers embedded liquid metal particles into electrospun polymer fibers (e.g., TPU), creating liquid metal nanofiber membranes (LMNMs). These membranes simultaneously achieve high elasticity (~400%) and superior moisture permeability (2941 g·m^−2^·d^−1^), ensuring prolonged comfortable skin contact. Sensors fabricated with this technology successfully monitored electromyographic (EMG) signals, validating their biocompatibility and the potential to be used as bioelectrode [69].

The liquid nature of metal nanoparticles also imparts high conformal adhesion, friction resistance, and sweat tolerance, making them promising for electronic tattoo technologies. Their seamless integration with skin textures ensures reliable performance during daily activities. Wang et al. developed a sprayable nanocomposite comprising MXene and liquid metal microcapsules. This material disperses liquid metal microcapsules (LM) and MXene nanosheets uniformly in a sodium alginate aqueous solution, forming electronic tattoos upon skin spraying. It exhibits exceptional friction resistance (only a 7% resistance increase after 40 finger-rubbing cycles) (Figure 3d,e) and maintains structural integrity and functional stability after 10 h immersion in tap water or artificial sweat (minimal resistance drift). Its motion artifact rejection capability directly results from seamless skin adhesion. The material enables dual-functional e-tattoos integrating sensing (e.g., ECG, EMG) and electrical stimulation functionalities, demonstrating substantial utility in wearable healthcare [70,71].

Liquid metal nanoparticles, combined with innovative structural designs and processing techniques (spray coating, stamping), overcome the failure modes of traditional metals under extreme deformation while significantly enhancing skin adhesion, environmental tolerance (e.g., friction/sweat resistance), and long-term stability. These advances substantially propel the development of flexible electrophysiological electrodes suitable for high-fidelity, long-term wearable dynamic physiological monitoring—particularly establishing a robust material foundation for e-skin and skin-integrated electronic tattoos.

Overall, the applications of solid metallic and liquid metallic nanomaterials exhibit distinct technical characteristics. Solid metallic nanomaterials (e.g., AgNW) leverage their high intrinsic electrical conductivity, mechanical strength, and functionalization potential to serve as core materials for static or low-frequency dynamic sensing scenarios. Through nanoscale structural regulation (e.g., oriented alignment, heterogeneous composite), these materials can construct ultra-thin flexible electrodes. For instance, aligning nanowire networks on electrospun substrates enables the fabrication of electrodes with high transmittance while maintaining low impedance and a high signal-to-noise ratio, meeting the requirements for high-precision physiological signal detection. However, their inherent brittleness limits applications under severe deformation, and long-term service often leads to fracture of the conductive network due to repeated mechanical stress. In contrast, liquid metallic materials (e.g., gallium-based alloy nanoparticles) transcend the deformation limitations of conventional metals through the combination of liquid properties and nanoconfinement effects. After nanoparticle formation, these materials can self-assemble or undergo interfacial engineering to form flexible conductive networks, maintaining conductivity even under ultra-large tensile strains (>600%) [68], and exhibiting self-healing capabilities that make them suitable for dynamic physiological monitoring and stretchable electronics. Nevertheless, their proneness to oxidation and challenges in interfacial stability increase process complexity, restricting large-scale application. Innovation in manufacturing processes is critical to translating the performance of both material classes. For solid metals, oriented growth techniques and substrate interfacial optimization are central strategies. For example, constructing oriented nanowire networks by regulating nanowire spacing and alignment optimizes carrier transport efficiency [72]. Additionally, interfacial engineering of composite materials can further enhance the comprehensive performance of electrodes. Liquid metal systems, on the other hand, rely on nanoparticle surface modification and macroscopic structural design to resolve the conflict between fluidity and stability. As previously noted, thermal sintering processes disrupt the oxide layer of nanoparticles and promote droplet fusion, forming continuous conductive pathways that maintain high elasticity while achieving ultra-high stretchability [68]; semi-embedded polymer fibers with confined structures physically restrict droplet migration, balancing conductivity and mechanical durability [69]. The synergistic development of these two material classes has driven significant advancements in physiological electrical sensing. The high intrinsic conductivity of solid metals and the superelasticity of liquid metals complement each other’s performance, while innovations in manufacturing processes—from nanoscale structural regulation to macroscopic interfacial design—enable gradient integration of material advantages. This progress expands the technical possibilities for next-generation smart electronic skins and implantable sensors.

To achieve the clinical application of bioelectrical sensors, advancements in device material performance must be complemented by breakthroughs in scalable manufacturing. While solid metals (e.g., gold, platinum) enable high-precision fabrication via traditional processes such as photolithography and sputtering, their high cost and low efficiency restrict application to small-batch precision devices (e.g., laboratory-grade neural electrode arrays). In contrast, liquid metals (e.g., EGaIn) can be mass-produced using injection molding, screen printing, and other techniques, significantly enhancing production efficiency. Nevertheless, challenges such as oxidation (surface formation of Ga_2_O_3_ passivation layers) and weak adhesion to rigid substrates remain barriers to large-scale implementation. The divergent properties of these materials highlight that solid metals are better suited for high-precision, small-scale scenarios, whereas liquid metals hold greater promise for scalable production. Non-metallic nanomaterials, such as carbon-based nanomaterials, offer dual processing advantages: they can undergo high-precision fabrication via in situ vapor deposition and be produced in batches through wet-process methods (e.g., screen printing, roll-to-roll forming). Beyond material processing, the translation of electrophysiological/electrochemical sensors from laboratory to clinical use requires careful consideration of biosafety, regulatory compliance, and ethical requirements. Implantable sensors must adhere to international standards such as ISO 10993 [73] (biological safety) and IEC 60601 [74] (electrical safety), with liquid metal devices additionally requiring accelerated aging tests to validate durability. Environmentally, liquid metals can be recovered via vacuum distillation, while solid metals should be manufactured using methods with reduced environmental impact. These considerations collectively provide clear pathways for addressing biosafety, regulatory, environmental, and clinical translation challenges.

This section systematically explores the properties of nanomaterials used in electrophysiological sensing and their biomedical application potential, with a focus on analyzing the differentiated advantages and limitations of non-metallic-based and metallic-based materials. Graphene, endowed with high electrical conductivity, ultra-thin flexibility, and excellent biocompatibility, has enabled multi-region synchronous high-resolution recording in cortical electrodes, while breakthroughs in the long-term implantation stability bottleneck have been achieved through h-BN encapsulation. MXenes, leveraging their transparent conductive properties and catalytic activity, have facilitated the construction of innovative electrode arrays that integrate synchronous optoelectronic signal acquisition and neural modulation functions. Among metallic materials, silver nanowire networks have realized the synergy of low impedance and antibacterial functionality through structural design, whereas liquid metal nanoparticles, with their exceptional elasticity and self-healing characteristics, have provided breakthrough solutions for stretchable electronic skins. Nevertheless, non-metallic-based materials still face challenges in long-term electrochemical stability; metallic nanostructures are susceptible to oxidation and inflammatory responses; and the high cost and large-scale fabrication difficulties of liquid metals remain unresolved issues. At the level of biocompatibility, while carbon-based materials exhibit natural tissue affinity, surface functional group modifications may induce nonspecific protein adsorption. Metallic materials require a balance between antibacterial performance and ion release toxicity—for example, long-term release of Ag^+^ from silver nanowires may induce local cytotoxicity. The biocompatibility of liquid metals remains controversial due to concerns regarding the gallium element, and their liquid nature may alter the interfacial microenvironment. Notably, interfacial engineering strategies significantly influence biocompatibility performance: graphene’s h-BN encapsulation effectively suppresses excessive activation of glial cells; MXene’s oxygen plasma treatment reduces interfacial impedance and fibrosis. Collaborative innovations in these materials have provided core support for next-generation high-spatiotemporal-resolution, multifunctional closed-loop neural interfaces. However, breakthroughs are required in material durability, interfacial modulation precision, and clinical translation costs to drive substantive advancements in the field of neural engineering.

### 2.2. Electrochemical Sensing Nanomaterials

The growing interest in developing devices for specific and sensitive quantification of biomarkers has positioned electrochemical sensors as ideal tools for disease detection and metabolic monitoring, owing to their low fabrication cost, portability, rapid response, and high sensitivity. Common detection methodologies include differential pulse voltammetry (DPV), cyclic voltammetry (CV), and square-wave voltammetry (SWV). Biosensors are fabricated through electrode modification: when biorecognition elements interact with target analytes, generated electrical signals are processed by electronic systems into quantifiable data. The sensitivity, selectivity, and stability of these sensors are fundamentally governed by electrode material design. Escalating demands for interference immunity, reusability, and fouling resistance are now driving material innovation as a critical breakthrough.

#### 2.2.1. Functionalized Materials

**Figure 4:**
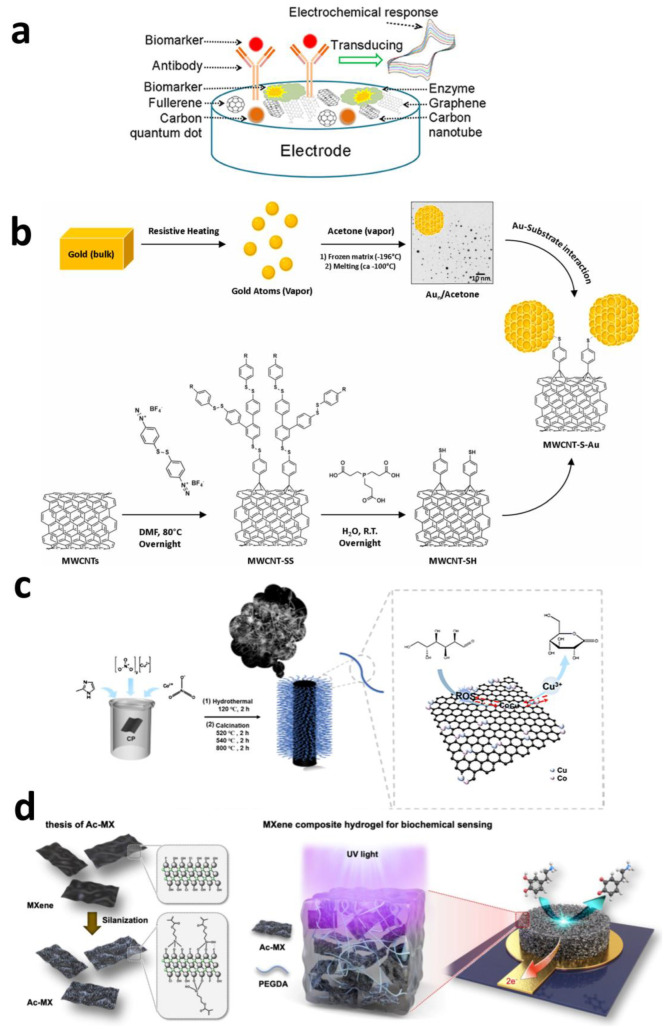
(**a**) A schematic representation of electrochemical biosensor [83]. Copyright © 2020, the author(s). (**b**) Schematic of the MWCNT-S-Au synthesis, illustrating the functionalization strategy to obtain MWCNT-SH based on a free radical reaction and disulfide bond cleavage, along with the synthesis of Au NPs via the MVS method and their grafting onto MWCNT-SH [89]. Copyright © 2024, the author(s). (**c**) Schematic illustration of the synthesis of CuCo-NCNTs [90]. Copyright © 2024, Elsevier. (**d**) Schematic of the synthesis of Ac-MX and the subsequent formation of a composite hydrogel [95]. Copyright © 2024, American Chemical Society.

The pursuit of biocompatibility, sensitivity, safety, and long-term performance for surgical instruments and implantable bioelectronic devices has continued for decades [75,76]. Among these, biocompatibility, particularly for long-term implantation applications, remains one of the most critical challenges [77,78,79]. The discovery of fullerenes in 1985 by Smalley’s team from Rice University and the University of Sussex [80], followed by the emergence of carbon nanotubes (1991) [81], graphene (2004) [82], carbon quantum dots, and nanodiamonds, revolutionized the field of materials science. These carbon nanomaterials, with their extraordinary intrinsic properties, offer revolutionary opportunities to overcome key limitations of implantable devices. Not only do they possess exceptional electrochemical characteristics—such as rapid electron transfer, wide electrochemical windows, significant capacitive behavior, and excellent chemical inertness—making them highly suitable for constructing electrical stimulation interfaces and sensor platforms, but they also feature soft surfaces, enhanced flexibility, a high surface-to-volume ratio (conducive to miniaturization), excellent tissue compatibility, and outstanding long-term in vivo stability. As shown in Figure 4a, this unique combination of advantages makes carbon-based materials an ideal choice for constructing next-generation high-performance implantable electrodes and electrochemical sensors, opening up broad prospects for their application in biomedical sensing [83].

Graphene, as a novel nanomaterial, has garnered considerable attention since its successful isolation. Furthermore, with advantages such as high electron mobility, high strength, and flexibility, it plays a vital role in biosensing applications. Athira Mani and colleagues developed a molecularly imprinted electrochemical sensor based on a composite of carboxylated graphene (GO-COOH) and gold nanoparticles (AuNPs) for the ultrasensitive detection of cortisol (Cor). GO-COOH provides a high specific surface area to enhance loading capacity, while AuNPs improve electron transfer efficiency, achieving a detection limit as low as 0.61 × 10^−14^ M. Utilizing the imprinting factor, the sensor exhibits excellent selectivity, effectively distinguishing Cor from interferents such as dopamine (DA) and cholesterol. The sensor maintains stable signals after 50 cycles and shows high recovery rates (96–101%) in human serum. Its greatest advantage lies in the synergistic combination of graphene’s high conductivity, AuNPs’ catalytic activity, and the specificity of molecular imprinting, enabling highly sensitive detection without requiring antibodies and offering a new strategy for portable health monitoring. Nevertheless, the preparation of GO-COOH involves a strong oxidation process that can introduce structural defects, leading to reduced intrinsic conductivity, necessitating reliance on AuNPs (15 ± 5 nm) to construct a conductive network for compensation. Additionally, GO-COOH still faces risks of partial reduction or aggregation during long-term use in physiological environments, requiring improvement through polymer coating and crosslinker optimization. While the core value of graphene lies in its tunable interfacial chemical properties, the complexity of the preparation process and long-term stability remain challenges for practical applications [84].

Md Asaduzzaman et al. constructed a highly conductive (~340 m^2^/g specific surface area) flexible substrate using laser-induced graphene (LIG) technology, modified with MXene@Pt/PtNPs, thereby avoiding the structural defects associated with strong oxidation processes. This platform was utilized for the detection of glucose, Na^+^ electrolytes, and pH in human sweat. The sensor exhibited high sensitivity (86.45 μA·mM^−1^·cm^−2^) and a low detection limit (3μM) for glucose, while the HNPC/PB composite structure expanded the detection range of the lactate sensor up to 100 mM. During 48 h of continuous testing, the signal attenuation was less than 5%, and it demonstrated excellent selectivity with less than 5% cross-reactivity to interferents such as lactate and ascorbic acid. The porous structure of the 3D graphene synergized with the catalytic activity of MXene@Pt and HNPC not only reduced the charge transfer resistance (from 115 Ω to 78 Ω) but also ensured reliable detection within the complex sweat matrix. The simplified fabrication process, employing a one-step chemical reduction synthesis method (NaBH_4_ reduction) for MXene@Pt and the direct laser writing process for LIG, significantly streamlined preparation compared to the complex oxidation–carboxylation procedure required for conventional GO-COOH while simultaneously preserving the tunable interfacial properties [85].

Compared to graphene, carbon nanotubes (CNTs) demonstrate greater flexibility and efficiency in signal transduction, surface functionalization, and biomolecular recognition within biosensing applications [86]. Electrochemical (EC) sensing approaches exploit CNTs as electrode materials owing to their unique structures and properties that impart strong electrocatalytic activity with minimal surface fouling. Nanofabrication and device integration technologies have emerged alongside significant advances in the synthesis, purification, conjugation, and biofunctionalization of CNTs. Such combined efforts have contributed to the rapid development of CNT-based sensors for a plethora of important analytes, offering enhanced detection sensitivity and selectivity. The use of CNTs provides an opportunity for direct electron transfer between enzymes and the active electrode area [87]. Due to their large surface area, high aspect ratio, excellent chemical stability, and extraordinary optical and electronic properties, carbon nanotubes are also widely employed in non-enzymatic sensor applications. Developing suitable or efficient electrocatalysts is crucial for producing highly efficient, inexpensive, sensitive, selective, and stable non-enzymatic sensors; these include metals (Au, Pt, Pd, Ni, Cu, Co, etc.), alloys, metal oxides (NiO, CuO, Co_2_O_3_, etc.), metal sulfides, and metal–organic frameworks (MOFs). Notably, coupling CNTs with numerous metals and metal oxides, such as nickel, copper, and copper oxides, exerts synergistic catalytic effects on glucose [88].

Giuseppe Misia modified multi-walled carbon nanotubes (MWCNTs) with ligand-free gold nanoparticles obtained via metal vapor synthesis. As shown in Figure 4b, this approach leverages the advantages of MWCNTs to enhance electron transfer and mass transport kinetics for a serotonin (5-HT) electrochemical sensor, yielding higher peaks and narrower full width at half maximum (FWHM). This improves the sensitivity and selectivity of voltammetric detection, achieving plasma serotonin detection with a sensitivity of 6.7 μA μmol^−1^ L cm^−2^ and a detection limit of 1.0 μmol L^−1^. A thin molecularly imprinted polymer layer was added to provide selectivity and anti-fouling properties to the sensor [89].

Beyond modification with single-metal particles, Zhenlu Zhao et al. designed a Cu-Co bimetallic active site nitrogen-doped carbon nanotube (CuCo-NCNTs) catalyst. As shown in Figure 4c, through electrodeposition modification combined with a programmed thermal treatment process, they achieved a significant enhancement in the electrochemical detection performance for glucose. Cu serves as the primary active site for glucose oxidation, while Co species, possessing excellent hydroxyl adsorption capacity, act as auxiliary sites that promote the catalytic reaction on Cu sites by continuously providing an oxygen source. This synergistic mechanism of dual active sites, combined with the unique micro-reaction environment of carbon nanotubes, endows CuCo-NCNTs with outstanding electrocatalytic performance: a sensitivity of 0.84 mA mM^−1^ cm^−2^ and a detection limit as low as 1μM, along with excellent stability and anti-interference capability [90].

Ruru Wang et al. modified a glassy carbon electrode (GCE) with a mixture of GO and MWCNTs, followed by further modification with a novel carbon composite and Au@Pt core–shell nanoparticles (Au@Pt NPs). The fabricated novel three-dimensional sensor interface was used for glucose detection, exhibiting a low detection limit (4.2 × 10^−8^ M) and two wide linear ranges: 5 × 10^−8^ to 1 × 10^−4^ M and 1 × 10^−4^ to 2.5 × 10^−3^ M [91].

MXene has recently gained prominence among two-dimensional (2D) nanomaterials. Its remarkable characteristics—such as high electrical conductivity, excellent hydrophilicity, good chemical stability, high surface area, tunable and abundant surface functional groups, facile large-scale synthesis in water, environmentally friendly properties, and non-toxic nature—endow it with bright application prospects in analytical chemistry. These include applications in fluorescence sensing, piezoresistive sensing, and photothermal sensing, particularly in electrochemical sensing. As a conductive 2D nanomaterial with a rich surface chemistry, MXene serves as an ideal carrier for biomolecules (e.g., enzymes, antibodies, or aptamers) in constructing electrochemical biosensors [92]. Relevant studies have also demonstrated MXene’s capability for simultaneous detection of multiple electroactive substances [93]. Despite these numerous positive attributes, MXene exhibits a strong tendency for inter-sheet aggregation, thereby compromising its electrochemical performance. To address this persistent challenge, researchers have sought to integrate MXene with other 2D nanomaterials, nanoparticles, enzymes, or 3D materials, forming composites with synergistic effects to enhance sensing performance [94].

As shown in Figure 4d, an advanced composite hydrogel combining MXenes and poly(ethylene glycol) diacrylate (PEGDA) enables the simultaneous electrochemical detection of neurotransmitters and antioxidants, addressing the critical interaction between oxidative stress and neurodegenerative diseases. MXene synthesis involves a mild etching route followed by functionalization with silane molecules to enhance activity and stability within the PEGDA hydrogel matrix. The composite hydrogel demonstrates exceptional antioxidant stability, surpassing its thin-film counterparts. The electrochemical sensor based on this composite hydrogel can detect multiple neurotransmitters with wide linear detection ranges: 2.5–200 μM for DA, 10–100 μM for UA, and 1–100 μM for 5-HT, achieving detection limits of 2.55 μM for DA, 25.11 μM for UA, and 0.83 μM for 5-HT. Compared to similar sensors, this system can simultaneously detect these multiple molecules in human serum [95].

**Figure 5:**
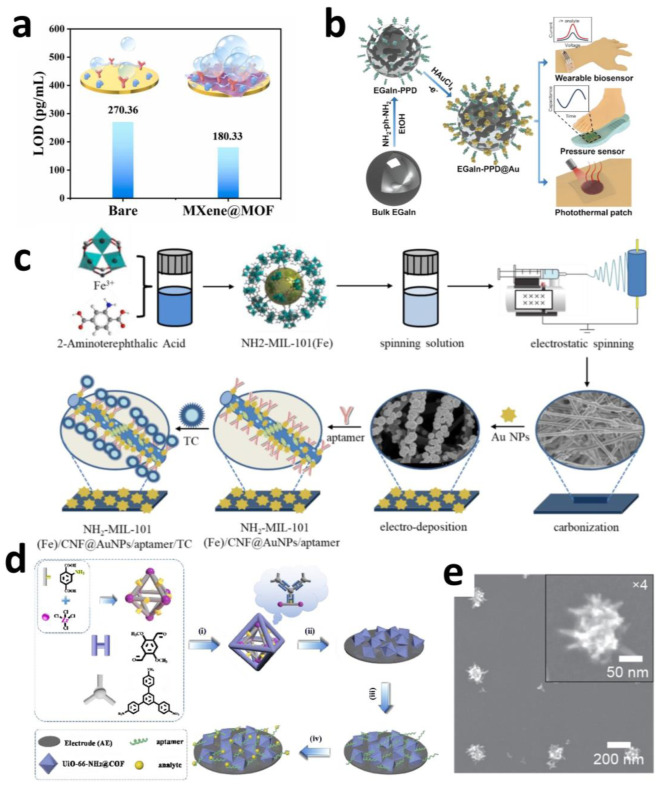
(**a**) Sensor sensitivity for detecting actual samples [96]. Copyright © 2020, the author(s). (**b**) Schematic drawing of the preparation of EGaIn-PPD@Au nanoparticles for various bioapplications [110]. Copyright © 2025, Elsevier. (**c**) Schematic illustration of the NH_2_-MIL-101(Fe)/CNF aptasensor preparation procedure [113]. Copyright © 2021, Elsevier. (**d**) Fabricating the MOF@COF-based electrochemical aptasensor for monitoring analytes. (**i**) Synthesis of UiO-66-NH_2_@COF. (**ii**) Deposition of UiO-66-NH_2_@COF. (**iii**) Immersed into aptamer (10.0 ng·mL^−1^) until the surface is saturated. (**iv**) Submerged in analytes for electrochemical tests [115]. Copyright © 2021, Elsevier. (**e**) SEM images of spiky (*a*_0_ = 600 nm; *d* = 150 nm) nanoelectrodes [121]. Copyright © 2025, American Chemical Society.

Glycoprotein non-metastatic melanoma protein B (GPNMB), as a potential biomarker for PD diagnosis and therapy, faces clinical application limitations due to its low abundance and the technical challenges posed by complex detection environments. Yindian Wang et al. developed an electrochemical biosensor based on an MXene@MOF composite material. As shown in Figure 5a, this sensor achieved the first label-free, ultrasensitive direct detection of GPNMB in the serum of PD patients. Through hetero-interface engineering, two-dimensional MOF materials were precisely loaded onto the MXene substrate, effectively inhibiting the oxidative degradation of MXene by H_2_O/O_2_. This conferred exceptional antioxidant stability, excellent conductivity, and a unique multi-layered folded structure to the material. These properties significantly enhanced the electroactive surface area of the sensor, enabling an extremely low detection limit (LOD) of 180.33 pg/mL. Clinical validation demonstrated the sensor’s outstanding discriminatory capability for PD patient samples (AUC = 0.91). This biosensor, combining high sensitivity, operational simplicity, and direct detection advantages, provides a powerful new technological platform for the early diagnosis, personalized treatment, and pathogenesis research of PD [96].

Despite these remarkable material innovations, practical implementation in flexible and implantable platforms still faces critical integration challenges. One major limitation is the mismatch in mechanical and electrical properties between high-performance sensing materials and flexible substrates [97], which can lead to delamination, microcrack formation, or signal instability during prolonged mechanical deformation. Additionally, while materials like graphene and CNTs offer excellent conductivity, maintaining stable electrical percolation within low-modulus or hydrated environments (e.g., hydrogels, elastomers) remains difficult [98]. Ensuring intimate interface coupling, reliable encapsulation, and scalable fabrication—such as solution-phase processing or printing-compatible formulations—will be essential for transitioning from laboratory demonstrations to clinical-grade devices [99].

#### 2.2.2. Metal Nanoparticles

To overcome the limitations of enzymes in biosensor applications, such as poor stability and short shelf life, researchers are actively seeking alternative materials with enzyme-like activity. In this context, metal nanoparticles (NPs) and metal oxide nanoparticles (MONPs) exhibit significant potential in the field of electrochemical biosensors due to their unique catalytic and electronic properties [100]. Metal NPs serve as efficient “electron wires” and “electrocatalysts,” significantly accelerating electron transfer processes. MONPs, with their excellent biocompatibility, are preferred materials for immobilizing biomolecules and constructing stable sensing interfaces. Additionally, semiconductor nanoparticles are commonly used for electrochemical labeling. Through various modification techniques (e.g., adsorption, deposition, bonding, polymerization), these nanoparticles can be firmly anchored to electrode surfaces, providing critical support for achieving high-sensitivity, high-stability, and miniaturized electrochemical biosensors [101].

Metal oxide nanomaterials play a pivotal role in electrochemical biosensors, enhancing the detection capabilities for specific analytes through their superior electrocatalytic performance. Cobalt oxides (e.g., p-type semiconductor Co_3_O_4_) are favored due to their high stability in strongly alkaline media (pH 13–14) and notable catalytic oxidation activity toward glucose and hydrogen peroxide. In alkaline environments, nickel electrodes form highly active Ni(III) oxyhydroxides on their surfaces, commonly used for amperometric glucose detection; under mildly acidic conditions (pH 5–6), the oxidative properties of nickel are also suitable for DA and ascorbic acid detection. To address synthesis scalability, recent reports highlight hydrothermal or sol–gel methods for batch fabrication of NiCo_2_O_4_ nanostructures under mild conditions, providing well-controlled morphology and reproducible electrochemical properties [102]. Combinations of cobalt and nickel (e.g., NiCo_2_O_4_) further enhance catalytic activity through synergistic effects between metals, proving particularly effective in glucose sensing [103,104]. Among copper-based materials, although copper nanoparticles can catalyze various molecules (e.g., glucose, hydrazine, hydrogen peroxide, dopamine), their susceptibility to poisoning by chloride ions, leading to deactivation, makes more stable copper oxides (e.g., CuO, Cu_2_O) a superior choice. In strongly alkaline environments, reversible transformation between copper oxide and copper hydroxide occurs, catalyzing glucose oxidation based on the Cu(II)/Cu(III) redox couple [105,106]. However, long-term stability remains a challenge—redox cycling and biofouling degrade performance. To counter this, researchers have introduced polymer coatings (e.g., Nafion, PVP) or hybridization with carbon supports (CNTs, graphene), which significantly improve the composite’s structural robustness and anti-fouling behavior in biofluids [107]. Iron-based oxides are extensively studied for mimicking enzymatic catalysis owing to their core role in natural peroxidases (e.g., heme iron-containing horseradish peroxidase); they primarily achieve enzyme-like catalytic behavior through Fe^3+^/Fe^4+^ redox cycling, commonly applied in H_2_O_2_ reduction and phenolic compound detection. Iron oxides are often composited with carbon-based materials (e.g., carbon nanotubes, graphene oxide), where the latter serves as a support scaffold providing large specific surface area, preventing aggregation, and significantly enhancing the conductivity and electron transfer efficiency of the composites [108].

Composites of gold nanoparticles and carbon materials also exhibit robust synergistic catalytic effects. For instance, gold nanoparticles immobilized on reduced graphene oxide (rGO) demonstrate activity far exceeding that of individual components. This enhancement stems from strong interactions between the orbitals of gold and carbon atoms, as well as Fermi level modulation induced by n-doping of rGO. To ensure compatibility with large-area flexible substrates, inkjet-printable metal nanoparticles and carbon materials hybrid inks have been developed, enabling scalable fabrication via additive manufacturing techniques [109].

To address the susceptibility of liquid metal EGaIn to oxidation, Mingyang Guan developed an EGaIn-PPD@Au composite material modified with gold nanoparticles (AuNPs). The synergistic interaction between PPD ligands and AuNPs not only resolves oxidation issues but also endows the material with exceptional electrical/thermal conductivity and photothermal properties. Wearable devices based on this material achieve high-sensitivity detection of C-reactive protein (0.5 mg/L). As shown in Figure 5b, its PDMS composite patch integrates precise pressure sensing (error < 0.003 MPa^−1^) with significant anti-inflammatory and pro-healing functions [110].

Electrode fouling is an inevitable phenomenon when encountering biological agents (e.g., dopamine, peptides, proteins), particularly during prolonged operation under oxidative contaminants. Diamond is emerging as an ideal antifouling sensing material due to its excellent fouling resistance and biocompatibility; however, its biomedical applications are constrained by limited detection sensitivity and specificity. In this work, a gold nanoparticle (Au-NPs)-reinforced nanoporous diamond sensing interface was innovatively developed. Au-NPs with uniform size (43.9 ± 12.7 nm) were evenly deposited via an electrodeposition process onto a nanoporous diamond substrate, significantly enhancing the interfacial electrocatalytic activity (heterogeneous kinetic constant reaching 6.7 × 10^−3^ cm s^−1^). This composite structure combines the stable anchoring and antifouling properties of nanoporous diamond with the enhanced electron transfer efficiency afforded by Au-NPs, substantially improving detection sensitivity for biomolecules such as dopamine. The synergistic effect between Au-NPs and a Nafion membrane enables highly selective DA detection: Au-NPs catalyze dopamine-specific reactions, while the Nafion membrane effectively screens out interferents like serum proteins. The sensor exhibits > 90% recovery rate (3–100 μM) in human serum and demonstrates exceptional signal stability (signal decay < 2.1% after one month, retaining 95% of initial response after six months) [111].

#### 2.2.3. Organic Framework Materials

MOFs are a recently emerging class of materials comprising self-assembled porous network-structured architectures formed by organic linkers connecting metal nodes. Consequently, MOFs—with their large surface area, controlled diverse pore structures, enhanced functionality, and unique catalytic activity—represent potential candidates for effective utilization as electrochemical sensors to detect biomolecules.

Li Zhao et al. developed a novel electrochemical sensing material, AuNR@ZIF-8, based on MOFs for the highly sensitive detection of neurotransmitters DA and serotonin (ST). Through precise regulation of ZIF-8 encapsulation, a core–shell nanostructure (average size ~175 nm) embedding gold nanorods (AuNRs) within ZIF-8 was constructed. This design combines the high specific surface area and abundant catalytic sites of MOF materials with the excellent conductivity of AuNRs. Benefiting from the porous confinement effect and synergistic catalysis of ZIF-8, the modified electrode exhibits significantly enhanced electrochemical performance: a linear detection range of 0.1–50 μM for DA (LOD 0.03 μM) and 0.1–25 μM for ST (LOD 0.007 μM). This study not only confirms the advantages of MOF-based composites in neurotransmitter detection but also provides a new strategy for developing efficient electrochemical sensors [112].

Jialing Song et al. fabricated a novel electrochemical aptasensor based on Fe-based MOFs, as shown in Figure 5c. To enhance sensitivity for antibiotic detection, NH_2_-MIL-101(Fe)/CNF@AuNPs was synthesized through a combination of hydrothermal, electrospinning, pyrolysis, and electrodeposition methods. Subsequently, aptamers could be attached to NH_2_-MIL-101(Fe)/CNF@AuNPs via “Au-S” bonds. The electrical signals generated by interactions between antibiotics and aptamers were amplified by the NH_2_-MIL-101(Fe)/CNF@AuNPs aptasensor, resulting in significant current/impedance responses. Thus, successful signal generation and amplification were achieved. The impedance detected by the sensor exhibited a linear relationship with tetracycline concentrations in the range of 0.1–10^5^ nM, with a minimum detection limit of 0.01 nM [113].

Compared to other materials, covalent organic frameworks (COFs) provide unique advantages for designing high-performance electrochemical biosensors due to their exceptional structural diversity, high specific surface area, and porosity. Their highly tunable structures allow for precise optimization for specific sensing requirements and can be readily modified with various functional groups or metal ions to develop sensors with high specificity. COFs also exhibit excellent chemical, mechanical, and structural stability, ensuring long-term sensor durability, while their biocompatibility makes them particularly suitable for biosensing applications. Furthermore, the good flexibility and film-forming capability of COFs demonstrate substantial potential for use in wearable and implantable sensors. These outstanding integrated properties have garnered significant attention for COFs in recent years, establishing them as strong candidate materials for developing novel electrochemical sensors [114].

Han-Wen Zhang combined MOFs and COFs to construct UiO-66-NH@COF composites through a covalent linkage approach, as illustrated in Figure 5d, where the microporous UiO-66-NH_2_ MOF core is encapsulated by the mesoporous TAPB-DMTP-COF shell. Crucially, the composite, which retains crystallinity and hierarchical porosity, significantly enhances electrochemical detection performance, such as for ATP and antibiotics. This is due to its high affinity between the aptamer’s phosphate groups and dense Zr(IV) sites, as well as strong π–π stacking interactions between the aptamer and this MOF@COF. The synthetic strategy in this systematic study extends the rational design of other MOF@COF core–shell hybrid materials to broaden their promising applications [115].

The issue of enzyme instability finds a solution within COF materials. Natural enzymes are directly encapsulated within hollow covalent organic framework (COF) capsules at room temperature. COF microcrystals migrate from the inner core and self-assemble on the outer wall, entrapping the enzyme within the cavity. The tunable hollow structure of the enzyme@COF capsules allows for essential vibrational freedom for the enzyme, significantly enhancing its biological activity. The hollow enzyme@COF capsules possess large mesoporous tunnels enabling efficient transport. Furthermore, the encapsulated enzymes within the capsules exhibit remarkable activity and ultra-high stability under various extreme conditions that could otherwise lead to enzyme denaturation. Finally, the prepared hollow GOx@COF capsules were utilized for the electrochemical sensing of glucose in human serum, with the electrochemical sensor demonstrating high selectivity and satisfactory test results. This novel approach to enzyme encapsulation within COFs holds potential applications in biocatalysis and biosensing, paving the way for artificial organelles.

#### 2.2.4. Anti-Biofouling Coatings

Implantable electrochemical sensing devices universally face electrode surface contamination by non-target substances (e.g., redox byproducts, biological metabolites) during prolonged detection, inevitably compromising sensitivity [116]. Anti-fouling coating technology serves as a critical strategy to mitigate such contamination [117]. By applying materials or microstructures that inhibit pollutant adhesion onto electrode surfaces, this approach significantly extends functional longevity in vivo. To preserve intrinsic detection activity, coatings must be ultra-thin or porous to ensure unobstructed analyte diffusion and charge transfer. Ideal anti-fouling coatings simultaneously provide electrochemical functionality, structural stability, biocompatibility, and long-term performance maintenance in complex biological environments.

Zhou et al. systematically compared multiple technical routes in their review of antifouling strategies: Conductive polymer functionalization (e.g., PEDOT-PC coatings) forms dense hydration layers via zwitterionic groups, extending the operational lifespan of carbon fiber microelectrodes in brain tissue from less than 1 h to several hours. Silica Nanoporous Membranes (SNMs) achieve size exclusion through ordered pores. Self-assembled Monolayers (SAMs) rely on amphifunctional molecules directly immobilized onto solid substrates to form antifouling layers. In situ electrochemical pulse technology, by contrast, cleans electrode surfaces via alternating potentials but does not directly enhance antibiofouling capability. Although each strategy has distinct advantages, the degradation of organic coatings in biological environments remains unresolved, limiting their application to short-term in vitro detection. Notably, this review also highlights that coating materials generally exhibit degradation issues, with typical operational lifespans of only several hours—though certain material types (e.g., electrochemical sensing electrodes modified with Au-terminated alkyne moieties) can function in biological environments for months. Nevertheless, these approaches are generally unsuitable for long-term recording due to severe immune response interference in biological media. Additionally, the biotoxicity of materials used in such methods requires further investigation [118].

Puthongkham et al. developed a nanodiamond (ND) coating that offers a novel strategy for carbon fiber microelectrodes. The sparse coating of 15 nm carboxylated ND used by the researchers reduced the charge transfer resistance to 0.30 Ω·cm^2^. Surface oxygen-containing groups in the coating selectively adsorbed cationic neurotransmitters, enhancing DA detection sensitivity by 2.1-fold (up to 29 ± 2 nA/μM) and optimizing the detection limit to 3 nM. Its hydrophilic surface (with a contact angle of 30°) significantly reduced protein adsorption, resulting in a signal retention rate of 64% after biofouling in brain slices—markedly higher than the 24% of bare electrodes. Notably, ND coatings with a thickness of 5 nm tend to form dense insulating layers that impede mass transport, underscoring the critical role of microtopography regulation in balancing sensitivity and antifouling properties [119]. Studies have demonstrated that ND exhibits the highest biocompatibility among carbon materials, particularly when particle diameters exceed 100 nm. Both in vivo and in vitro experiments confirm the non-toxicity of ND; however, large ND particles pose challenges due to in vivo accumulation and inadequate metabolic excretion [120].

However, such coating-induced material alterations may compromise precision electrochemical sensors. To achieve effective anti-fouling without modifying bulk materials, Wu et al. developed nanospike architectures that circumvent traditional coating limitations. Gold spike arrays (Figure 5e) with tip radii < 10 nm (300 nm periodicity) fabricated on ITO substrates demonstrated intrinsic anti-fouling properties, exhibiting 5.8-fold greater signal retention in human serum than planar electrodes. This work provides novel design insights for surface structuring in implantable sensors. Nevertheless, this study only conducted bacterial mRNA detection in vitro and did not thoroughly investigate the biocompatibility or metabolic processes associated with this nanostructure [121].

Recent trends in electrochemical sensor anti-fouling coatings reflect a paradigm shift from singular material modifications to structural–functional synergy. Early research emphasized surface chemical modifications, (e.g., hydrophilic groups creating anti-adhesion barriers), yet these layers remain vulnerable to biological degradation and will suffer a certain degree of damage during continuous operation, leading to a decline in the anti-biofouling ability. However, coating materials still present limitations by altering the detection interface; those fabricated from inactive materials can obscure the active surface, while excessively thick coatings may further impede charge/mass transport, thereby compromising sensing performance. The nanospike strategy involves micro/nano-topological modifications to base materials, leveraging geometric effects to optimize electron transport pathways and reduce steric hindrance, thereby achieving a synergistic enhancement in both anti-biofouling properties and sensitivity. However, the protruding microstructures are prone to mechanical damage (e.g., abrasion, detachment) under high-strain and high-shear operational environments. Furthermore, detached spiked nanostructures may exert adverse effects on biological tissues. Pending comprehensive validation of biocompatibility, such configurations are not yet suitable for direct implementation as structural components in implantable devices. These progressively evolving anti-biofouling strategies have collectively propelled the practical implementation of implantable sensors in complex biological environments, thereby providing critical technical enablers for long-term dynamic monitoring. Future efforts could focus on the synergistic integration of the aforementioned strategies, leveraging biocompatible materials to construct anti-fouling microstructures. This approach is anticipated to achieve comprehensive enhancements in biocompatibility, operational lifespan, and detection performance.

### 2.3. Structural Design for Performance Enhancement

The bonding mechanisms and microstructural organization between constituent phases in composite materials directly influence material properties. For biosensing electrodes, these structural features critically impact mechanical behavior, electrical characteristics, sensing capabilities, and long-term stability. Targeted microstructural design thus enables precise tuning of specific properties to meet sensor performance requirements.

#### 2.3.1. Mechanical Microstructure Engineering

Long-term electrophysiological monitoring necessitates mechanical robustness in dynamic physiological environments (e.g., stretching, shearing, torsion) while preventing signal distortion or tissue damage caused by modulus mismatch [34,122]. This demands materials exhibiting low Young’s modulus [123], high stretchability (>200% strain) [124], and exceptional fatigue resistance [125]. Recent breakthroughs through microstructural innovations are reviewed below, focusing on three nanoscale strategies for mechanical enhancement.

Incorporating nanonetwork structures represents a direct approach to augment mechanical properties, where porous scaffolds simultaneously enable stress distribution, ultra-thin configurations, and breathability. Zhang et al. engineered a 10 μm thick gelatin hydrogel reinforced by a polyurethane (PU) nanomesh. Functioning as a reinforcing skeleton, the nanomesh (density: 0.3 mg·cm^−2^) was embedded within a thermoresponsive phase-transition gel, significantly boosting mechanical strength. This composite achieved a tensile strain of 696%, toughness of 44.8 kJ·m^−3^ (compared to 7.3 kJ·m^−3^ for unreinforced gels), and endured 1000 cycles at 100% strain.

The material’s ultra-thin architecture and porous structure conferred skin-equivalent breathability, with moisture transmission exceeding 90% of bare skin levels, effectively preventing inflammatory responses during extended wear. This design overcomes the gas transport limitations inherent in traditional thick hydrogels (>1 mm thickness), establishing a foundational approach for enhancing comfort in wearable biomedical devices [126].

**Figure 6:**
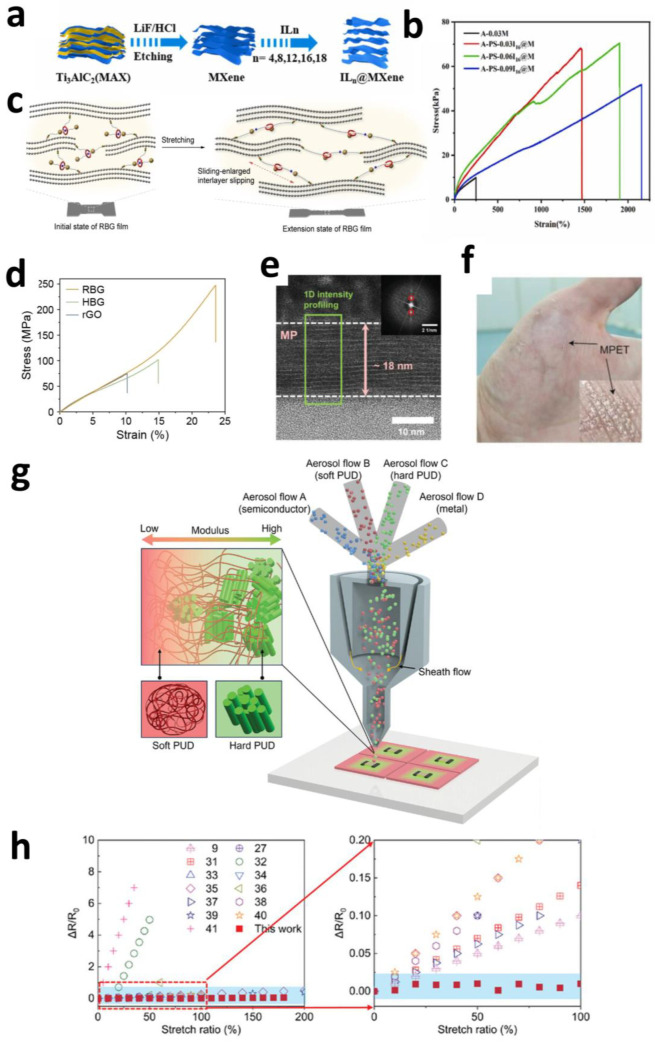
(**a**) Schematic of the synthesis of MXene and IL_n_@MXene. (**b**) Hydrogels with different contents of I_16_@M [127]. Copyright © 2024, Elsevier. (**c**) The hidden chain of [2]rotaxane was released due to intramolecular motion when applied force, resulting in an increased interlayer slip distance. (**d**) Typical stress–strain curves for rGO, HBG, and RBG films [128]. Copyright © 2024, Wiley-VCH. (e) HRTEM images of a cross-sectional view of MP. (**f**) MPET electrode on the hand [130]. Copyright © 2023, Wiley-VCH. (**g**) Scheme for one-step AMMP to print both UGI and functional devices. (**h**) Comparison of RRC in this work with previously reported stretchable electronics [132]. Copyright © 2025, the author(s).

In MXene/hydrogel composites, strategic intercalation agent incorporation optimizes two-dimensional material dispersion and interfacial stress transfer efficiency. Zhang et al. discovered ionic liquid (IL) intercalation effectively suppresses MXene self-restacking, with maximal interlayer expansion occurring at an alkyl chain length of C = 16 (Figure 6a). Integration with poly(acrylic acid) (PAA) networks and polydopamine-modified silica (PS) yielded an A-PS-0.06I_16_@M hydrogel system. Within this architecture, MXene serves as the conductive phase, while Polydopamine (PDA) @SiO_2_ microspheres function as the adhesion-enhancing component; hydrogen bonding and electrostatic interactions among functional groups (–OH, –F) collectively reinforce the polymer matrix. This synergistic design achieves a tensile strain of 1903% (Figure 6b), skin-matching low modulus (20 kPa), and exceptional toughness (806 kJ·m^−3^). By fundamentally resolving the mechanical degradation issues caused by aggregation-prone 2D materials, this methodology establishes novel design principles for highly ductile conductive hydrogels [127].

Nanomaterial interfaces can be reinforced through dynamic molecular bridging strategies that exploit mechanical bond sliding to dissipate energy and enhance mechanical properties. Wang et al. integrated [2]rotaxane as interlayer bridging units between graphene sheets (Figure 6c). Under external stress, the sliding motion of [2]rotaxane molecules releases concealed chains, increasing interlayer slip distances. This yields an unprecedented fracture strain of 23.6% (versus 10.2% in unmodified counterparts) and exceptional toughness of 23.9 MJ·m^−3^ (a 6-fold improvement over controls). In conventional layer-stacked graphene films, nanosheets slide under load, yet weak interlayer interactions (primarily hydrogen bonding) constrain slip distances to <10 nm, causing mechanical deficiencies. The mechanical bond mechanism fundamentally addresses this limitation: intramolecular sliding of benzo-24-crown-8 (B24C8) wheels along secondary ammonium salt axles enables slip distances >50 nm. Consequently, the resulting film simultaneously achieves 247.3 MPa tensile strength and high ductility (Figure 6d), overcoming graphene’s intrinsic strength–toughness trade-off. This molecular engineering approach demonstrates significant potential for advanced flexible bioelectrodes [128].

Diverging from conventional polymer blending methodologies, three distinct structural design strategies—nanonetworks, intercalation doping, and molecular bridging—collectively address the persistent strength–ductility trade-off in flexible electronics: Nanonetworks establish three-dimensional frameworks for isotropic stress distribution; intercalation doping optimizes nanomaterial dispersion and interfacial interactions; molecular bridging exploits dynamic bond reconfiguration for efficient energy dissipation. These paradigm-shifting mechanisms synergistically propel the advancement of durably stable bio-integrated electronic systems.

#### 2.3.2. Electrical Microstructure Design

The electrical performance of bioelectrodes is governed by two primary factors. First, intrinsic material properties: resistance variations under mechanical deformation can critically compromise signal transmission and generate motion artifacts that distort recorded signals [129]. Enhancing conformal contact at bio-interfaces and improving conductivity can partially mitigate motion-induced interference.

Nanomaterial-enabled device ultra-miniaturization and structural densification enhance interfacial conformality and conductivity, effectively suppressing motion artifacts. Song et al. developed an ~20 nm ultra-thin MXene (Ti_3_C_2_T_x_) film electrode (MPET) (Figure 6e). Leveraging its nanoscale thickness and resultant interfacial conformality, the MPET electrode achieves significantly reduced contact impedance with skin or neural surfaces (~10 kΩ@10 Hz, ~5 kΩ@100 Hz). During the detection of electrophysiological signals—including EMG, electroencephalography (EEG), electrooculography (EOG), and electrocardiography (ECG)—MPET exhibits exceptional SNR and signal-to-motion artifact ratios (SMR), particularly under dynamic conditions. In multichannel facial EMG monitoring, the MPET array precisely discriminates bite force magnitudes across distinct anatomical locations, while commercial gel electrodes suffer adhesion loss during movement and lack spatial resolution (Figure 6f). This performance superiority stems from MPET’s ultra-thin conformation eliminating interfacial mechanical slippage through intimate skin coupling, thereby suppressing motion artifact generation [130].

Beyond device miniaturization, designing intelligent interfaces between electrode materials and soft biological tissues/substrates through optimized microstructural organization represents a pivotal strategy for resolving stiffness mismatch and achieving strain-insensitive electrical performance [131]. This approach significantly expands the applicability of nanomaterials with superior electrical properties but limited mechanical robustness.

Song et al. implemented a three-dimensional universal gradient interface (UGI) (Figure 6g) with submicron resolution using aerosol multi-material printing (AMMP). By precisely modulating spatial distributions of soft and rigid components within a polyurethane substrate, UGI establishes microscale stiffness gradients. When external stretching (e.g., 100% uniaxial strain) occurs, UGI’s strain-insulating effect confines deformation in the top rigid region to negligible levels (<1.1%). Consequently, brittle high-performance electronic materials (Au nanoparticles, Ag nanowires, MXene, MoS_2_) deposited on UGI’s rigid zones exhibit near-invariant resistance (<1.9% relative change) (Figure 6h) even under extreme deformation (180% uniaxial strain). This microscale stiffness–gradient architecture fundamentally resolves electrical instability issues for rigid functional materials on soft, dynamic substrates.

Researchers directly printed multifunctional pulse oximeters integrating MoS_2_ photodetectors, LEDs, and MXene strain sensors onto UGI. Leveraging exceptional strain isolation, photocurrent signal drift remained below 1.7% during simulated motion (20% artificial skin strain), demonstrating near-perfect motion artifact immunity. In contrast, sensors printed directly on soft substrates exhibited >547% signal variation. This artifact resistance and compatibility with diverse high-performance materials (semiconductors/metals) highlight UGI’s transformative potential for next-generation high-fidelity, wearable/implantable bioelectronics including real-time health monitors and neural interfaces [132].

Beyond intrinsic material resistivity, bioelectrode interfacial impedance critically impacts electrical performance by degrading signal quality and precision. Elevated impedance reduces SNR, severely compromising recording fidelity. Most electrode materials cannot simultaneously achieve electronic conductor-level conductivity and ionic conductor-level low interfacial impedance—a capability primarily limited to mixed ionic-electronic conductors (MIECs) like PEDOT:PSS [133]. Conventional approaches apply PEDOT:PSS as surface coatings [78,134] or dopants [135]. While moderately effective, these strategies face fundamental limitations imposed by MIECs’ inherent properties. Without superior MIEC alternatives, performance improvements approach theoretical ceilings. Alternatively, structural engineering of interfacial materials can emulate MIEC functionality: enhancing conductivity while reducing impedance.

**Figure 7:**
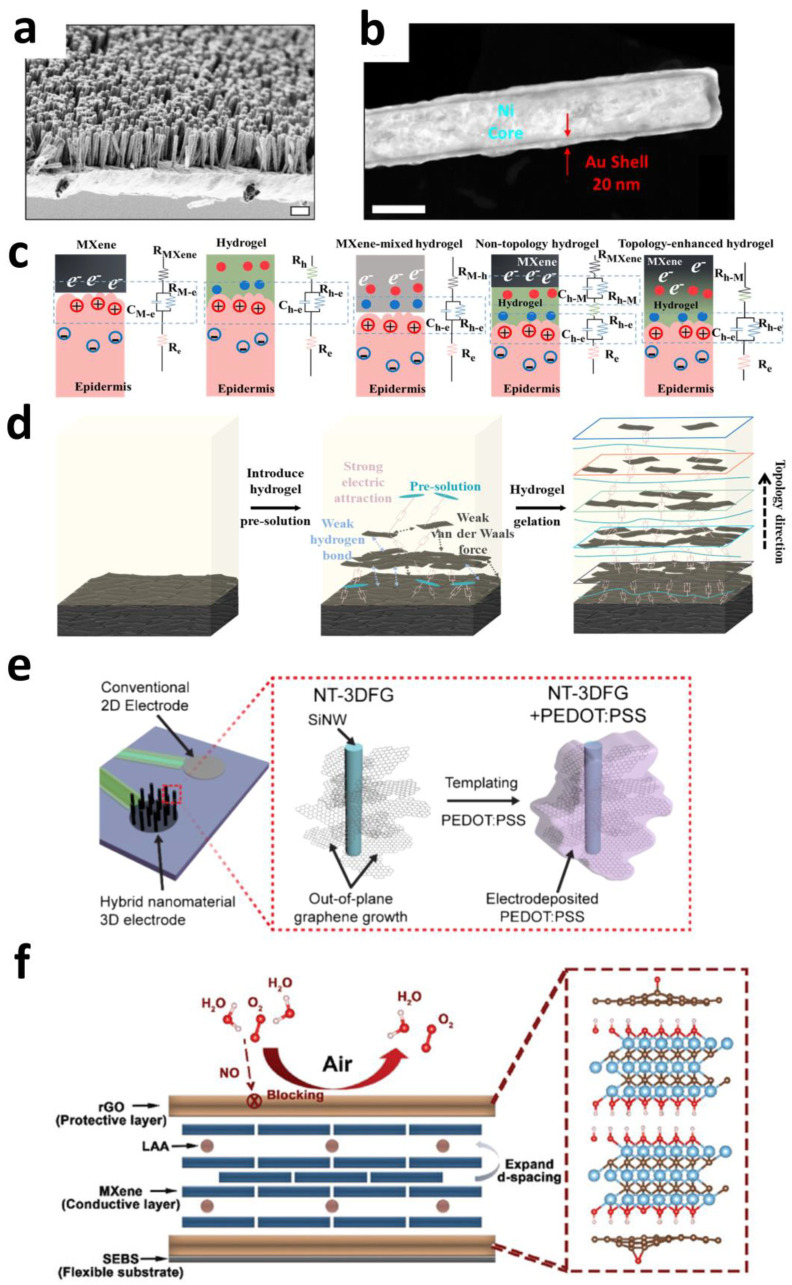
(**a**) Representative SEM images of Ni–Au core–shell cross-section. Scale bar: 1 µm. (**b**) Representative TEM Z-contrast images of free-standing Ni–Au NWs detached from the electrodes. Scale bars: 100 nm [137]. Copyright © 2024, the author(s). (**c**) Equivalent circuit model of MXene, the hydrogel, MXene-mixed hydrogel, non-topology hydrogel which forms a MXene/hydrogel double layer, and topology-enhanced hydrogel, which explains the effect of the topological MXene network. (**d**) Formation process of the topological MXene network. Copyright © 2024, American Chemical Society [138]. (**e**) Schematic illustration of a conventional 2D and multidimensional nanostructured NT-3DFG electrode and synthesis of a hierarchical hybrid nanomaterial by conjugating PEDOT:PSS on NT-3DFG [143]. Copyright © 2024, the author(s). (**f**) Schematic structure of rGM thin film for AED electrodes [146]. Copyright © 2025, Wiley-VCH.

A traditional method for impedance reduction involves increasing electrochemically active surface area using disordered porous materials [136]. Advanced fabrication techniques now enable precise engineering of ordered nanostructures, permitting meticulous control over both active surface dimensions and morphological configurations. Rodilla et al. developed an innovative flexible metal core–shell nanowire electrode, significantly improving electrical performance and biocompatibility (Figure 7a,b). Using template-assisted electrodeposition, they fabricated vertically aligned Ni-Au core–shell nanowire arrays as neural interface active surfaces. The nickel core provides mechanical stability, enabling exceptionally long and uniform nanowires (~1.9 μm length with >3-fold reduction in standard deviation). Complete gold encapsulation ensures biocompatibility and chemical stability. This hierarchical structure reduces electrode impedance 9-fold compared to planar gold electrodes (9.6 ± 0.6 kΩ compared to 83 ± 25 kΩ)—primarily due to an 8.4-fold increase in electrochemically active surface area relative to the geometric area. This core–shell design simultaneously resolves three challenges: overcoming single-metal nanowire limitations (e.g., pure Au), preventing biocompatibility issues from nickel exposure, and achieving significant impedance reduction via enlarged surface area. It provides a robust microstructural solution for high-performance flexible neural electrodes [137].

Diverging from surface–microstructure strategies for enlarging active areas, Luo et al. proposed an interfacial engineering approach based on topological MXene networks (Figure 7c,d). By constructing gradient topological interconnects between conductive MXene layers and ion-conducting hydrogels, this architecture optimizes interfacial charge transfer mechanisms. Critically, this configuration constitutes neither simple physical mixing of MXene particles within hydrogels nor mechanical bilayer stacking of MXene films with hydrogels. Instead, a specialized fabrication process partially delaminates MXene from stacked lamellae, forming molecular-scale interpenetrating networks with hydrogel prepolymer solutions through strong electrostatic attraction. This structure reduces interfacial impedance via three synergistic mechanisms: the topological MXene network enhances bulk dielectric properties; extended Debye length promotes uniform ion distribution and broadens the electric double layer region; and localized electric fields surrounding MXene become significantly intensified, enhancing charge transfer efficiency. Collectively, these effects enable the topology-engineered hydrogel to achieve ultralow through-thickness impedance (<20 Ω across 10^2^–10^5^ Hz)—11.4-fold lower than non-topological counterparts. At 1 V applied potential, DC output increases 7.7-fold while exhibiting smaller and more stable polarization potentials. Implemented in multichannel flexible bioelectronics, this hydrogel sustains high-fidelity, low-noise ECG recording with an exceptional signal-to-noise ratio while maintaining a stable performance for over 60 days, thereby providing a potentially universal solution for optimizing interfacial charge transport [138].

These two distinct approaches—employing ordered core–shell nanowires and gradient topological networks—have demonstrated substantial improvements in interfacial performance. This evidence confirms that innovative microstructural engineering strategies targeting electrode–bio-interface architectures hold large potential for advancing electrophysiological sensor capabilities. Collectively, they establish foundational design principles for developing next-generation high-performance neural interfaces and bioelectronic systems with minimal interfacial impedance.

#### 2.3.3. Sensing Microstructures

To enhance electrode sensing precision and optimize interfacial performance with biological tissues, nanoparticle modification or intricate microstructure design of electrode surfaces has been extensively investigated. These nanoscale features significantly increase the electrode’s effective active surface area, optimize charge transfer characteristics, reduce interfacial impedance, and introduce unique physicochemical properties, thereby improving the electrode’s capability to capture weak physiological signals (e.g., neural action potentials, electromyographic signals) and ensuring long-term recording stability.

Metal nanoparticles exhibit outstanding catalytic activity due to size-induced valence electron effects (phenomena arising from reduced particle size, which alter electronic properties by confining valence electrons within nanoscale dimensions) [139], playing crucial roles in electrochemical sensing. However, their dispersed state inherently limits conductivity, typically necessitating application as surface modifiers or additives adhered to or blended into electrodes. This relatively crude utilization method frequently leads to surface particle deactivation (e.g., oxidized surface), detachment, and occlusion of catalytic sites (e.g., diffusion restrictions), severely constraining functionality.

Appropriate structural design can optimize the performance of metal nanoparticles. Chen et al. developed a Bi_13_S_18_I_2_/Bi_2_WO_6_ double-interlocked heterojunction (a nanostructured material combining two semiconductors in an intertwined lattice to synergize band structures and photophysical properties) electrode that covalently immobilizes Au nanoparticles on high-surface-area substrates via click chemistry (N_3_-DBCO), achieving stable loading and high-density integration. This architecture facilitates efficient capture of active carriers generated by Au nanoparticle local surface plasmon resonance (LSPR) effects, while the heterojunction’s photothermal-band structure synergy creates directional driving forces. Consequently, Au nanoparticle catalytic performance is enhanced. The resulting flexible Glu sensor demonstrates a detection limit of 0.02 pM and an initial photocurrent surge of 15 μA·cm^−2^, validating the core value of structural design for optimizing nanoparticle catalysis. This approach provides a feasible strategy for photoelectrochemically enhanced sensing capabilities [140].

Metal nanoparticles additionally enhance electrode electrophysiological detection capabilities. Ramezani et al. developed a high-density electrode array using platinum nanoparticle-decorated intercalation-doped double-layer graphene named as PtNP/id-DLG (i.e., Pt atoms inserted between graphene layers to expand interlayer spacing), overcoming the quantum capacitance limitation (i.e., a fundamental constraint on charge storage density imposed by the Pauli exclusion principle) of graphene and significantly increasing charge storage capacity. After 150 s electrochemical deposition of PtNPs on 20 μm diameter electrodes, impedance decreased substantially while charge storage capacity increased 7.5-fold (from 5.4 ± 1.1 MΩ to 250 ± 56 kΩ @1 kHz). The transparent 256-channel array successfully synchronized cortical surface potential recording with two-photon calcium imaging in vivo. Its small size and low impedance characteristics enabled the capture of multi-unit activity (MUA) high-frequency signals, and it can decode single-cell calcium activities from surface potentials via neural network models with cellular-scale spatial resolution [141].

Beyond functional metal nanoparticles, specialized structural designs can augment electrodes’ sensing capabilities. Lee et al. fabricated vertically aligned ZnO nanotubes (500 nm diameter, 7 μm height) with uniform positioning and controlled morphology using metal-organic vapor phase epitaxy (MOVPE). After Ti/Au metallization, this architecture achieved an impedance below 100 kΩ at 998.26 Hz and 80% visible-light transmittance (450 nm wavelength), enabling dual-function electrophysiological recording and fluorescence imaging (i.e., simultaneous electrical recording of neural activity and fluorescence imaging of cellular structures). The system successfully captured intracellular-like potentials (2–4 mV amplitude) and extracellular spikes (0.5–1.5 mV) from mouse hippocampal neurons while concurrently visualizing neuronal somata and synaptic connections through calcium imaging. This approach provides a novel solution for synchronized electro-optical monitoring of neural circuits [142].

Ranke et al. engineered a composite material integrating hierarchical nanowire-templated three-dimensional fuzzy graphene (i.e., a string of graphene with planes perpendicular to the central axis of the nanowires) named NT-3DFG with PEDOT:PSS (Figure 7e). Conformal PEDOT:PSS coating on vertically aligned graphene sheets within NT-3DFG yielded a 30-fold enhancement in CIC compared to platinum electrodes. This architecture enables DA detection through amplified electrochemical activity while leveraging edge catalytic sites to selectively generate H_2_O_2_ for neuronal microenvironment regulation, thereby establishing foundational capabilities for closed-loop neuromodulation strategies [143].

Multidimensional nanomaterial hybridization and microstructural design significantly optimize biosensor performance by enhancing charge injection capacity and improving detection sensitivity. These structural strategies transcend intrinsic material limitations, providing foundational design principles for multimodal neural interface microarchitectures. Future research may explore the combinatorial integration of diverse nanomaterials, presenting opportunities for achieving comprehensive performance enhancement in next-generation electrodes.

#### 2.3.4. Stability-Enhancing Microstructures

Certain high-performance electrophysiological materials, such as the emerging two-dimensional MXene, are prone to oxidation through reactions with environmental water and oxygen, leading to significant conductivity degradation and structural deterioration (e.g., delamination or fracturing of nanosheets) [144]. This severely limits their application in bioelectrical interfaces requiring long-term operational stability. Nanostructural design strategies—such as introducing functional groups or constructing protective layers on electrode surfaces—can effectively isolate materials from environmental erosion or reduce reactive sites, thereby substantially enhancing material stability and device longevity.

Incorporating protective substances to passivate reactive sites represents the most direct protection method for oxidation-prone materials. Taking MXene as an example, Chae et al. improved its aqueous oxidation resistance through catechol-functionalized surface modification. The study revealed that catechol-functionalized polyvinyl alcohol (PVA-CA) forms strong bonds with MXene surfaces via hydrophobic benzene rings, significantly reducing oxidation-active sites. This protective mechanism arises from the hydrophobic barrier effect of catechol groups, which block water molecule access to MXene’s active edges, thereby significantly delaying the oxidation process [145].

Constructing physical barriers resembling core–shell structures on oxidation-prone materials represents an alternative approach to isolate reactive substances (e.g., water, oxygen). Xiong et al. developed a reduced graphene oxide (rGO) overlayer as a protective barrier to suppress MXene oxidation. Through in situ reduction of GO by L-ascorbic acid at 150 °C while forming Ti-O-C bonds with MXene surfaces, they fabricated rGO@MXene heterostructures (denoted rGM) (Figure 7f). After 40-day air exposure, the sheet resistance of rGM increased by only 130%, compared to a 1383% increase for pristine MXene films. This protection mechanism originates from two synergistic effects: charge redistribution creates a built-in electric field (BIEF) that accelerates charge transfer, while the isolation layer reduces water molecule adsorption energy through enhanced surface hydrophobicity, thereby significantly delaying oxidation kinetics [146].

To address the susceptibility of MXenes to environmental oxidation in bioelectronic interfaces, where reactions with water/oxygen lead to conductivity degradation and structural collapse, current research has proposed two primary mitigation strategies: chemical passivation and physical barrier construction. The chemical passivation strategy, characterized by low cost and facile processability, is compatible with devices operating in aqueous environments; however, a drawback is that the passivation layer may moderately compromise electrode conductivity. In contrast, the physical barrier strategy offers advantages of high stability and a broad operating temperature range, making it suitable for both wearable and implantable bioelectronic devices, though its limitation lies in the potential impact of the barrier layer on interfacial performance. These two approaches inhibit oxidation kinetics through chemical bonding and physical isolation, respectively, providing key technical support for the development of air-sensitive bioelectronic devices (e.g., implantable brain–computer interfaces) that combine high conductivity with long-term stability.

### 2.4. Multimodal Integrated Sensing Devices

Traditional electrochemical biosensors rely on a single signal output, rendering them susceptible to environmental interference and complex sample matrices. Although existing techniques (e.g., biometrics, ratiometric readouts, machine learning) have attempted to mitigate this issue, they remain constrained by the inherent limitations of the single detection modality itself [147].

Several key signal transduction mechanisms are foundational to multimodal biosensing. Ratiometric fluorescence involves the simultaneous measurement of two fluorescence peaks (for example, at 441 nm and 550 nm), and the use of their intensity ratio allows for internal calibration that compensates for probe concentration and environmental variability. Photothermal sensing relies on detecting the heat generated when a target analyte absorbs light at a specific wavelength, which is registered as a measurable temperature change. Electrochemiluminescence (ECL) refers to light emission produced by electrochemical reactions occurring at an electrode surface. Photoelectrochemistry (PEC) involves the generation of an electrical signal upon light irradiation of a semiconductor electrode, enabling sensitive detection in photoactive systems.

Inspired by the human tongue and nose as differential sensing systems, where simultaneous cross-reactive interactions between multiple analytes and sensor units create unique patterns or fingerprints for each analyte, such systems enable the discrimination and identification of analytes with similar structures or complex mixtures of unknown structures/components [148]. This biological paradigm demonstrates how multiple receptor channels combine to improve selectivity and robustness: when one channel’s response is perturbed (e.g., by pH shifts or background interference), other channels compensate, forming a redundancy-check mechanism that enhances overall reliability. Drawing from this principle, multimodal biosensors integrate two or more distinct signal transduction mechanisms (e.g., electrochemical, optical, etc.). Through cross-verification of multiple signals, they significantly reduce false positives/negatives, compensate for the sensitivity or adaptability shortcomings of any single modality, and enhance overall detection stability and flexibility for complex biological samples.

Under major infectious disease threats such as the COVID-19 pandemic, early and precise detection is paramount. Analogous to how integrating multiple technologies improves accuracy in clinical diagnostics, developing electrochemical-based multimodal/multi-signal sensing technologies enables the synthesis of information from diverse biochemical indicators. By generating more reliable results through logical or comprehensive judgment, this approach holds significant importance for addressing public health challenges and advancing personalized medical diagnostics [23].

#### 2.4.1. Combination of Electrochemical with Optical, Visual, or Thermal Sensing Technologies

Electrochemical (EC) sensing technologies detect and quantify analytes based on their electrochemical responses, encompassing diverse principles such as amperometry, voltammetry, potentiometry, impedance, conductometry, photoelectrochemistry (PEC), and ECL, each offering distinct advantages and application scenarios. To overcome the limitations of single-signal detection, multimodal biosensors based on multimodal nanomaterials (integrating superior optical, electrical, magnetic properties, etc.) have emerged. These sensors utilize multiple relatively independent detection mechanisms and signal transduction processes (e.g., combining EC, optical, magnetic signals, etc.) to achieve multiple signal outputs. This strategy not only reduces interference but also significantly enhances the reliability of detection results through mutual verification among signals.

A ratiometric fluorescence–colorimetric dual-mode sensing system based on enzymatic cascade activation (AOX/CeO_2_@TPE + OPD) was developed by embedding an aggregation-induced emission (AIE) molecule, TPE, into hollow CeO_2_ nanospheres, thereby constructing a blue-fluorescent nanozyme CeO_2_@TPE with high peroxidase-like activity. This system utilizes CH_3_SH to trigger a two-step cascade: first, activating alcohol oxidase (AOX) to catalytically convert CH_3_SH into H_2_O_2_, which subsequently stimulates CeO_2_@TPE to generate •OH radicals that oxidize o-phenylenediamine (OPD) into yellow 2,3-diaminophenazine (DAP). This process yields a distinct absorbance peak at 425 nm and induces a secondary fluorescence emission at 550 nm, complementing the intrinsic 441 nm emission of CeO_2_@TPE to form a robust ratiometric fluorescence output. The dual-mode readout—colorimetry (visible to the naked eye) and fluorescence (quantitative precision)—is further enhanced by integration with a smartphone-based analytical platform for on-site, real-time detection of CH_3_SH via digital hue parameter analysis. Importantly, the system demonstrated high analytical performance, achieving a detection limit as low as 80.3 nM (3.86 ppb) in fluorescence mode and 145.9 nM (7.01 ppb) in colorimetric mode, with wide linear ranges up to 200 μM and excellent anti-interference capability. In real-world validation, the sensor achieved recovery rates between 96.00% and 106.00% and relative standard deviations under 5% in untreated municipal, hospital, and canteen sewage samples, confirming its reliability, reproducibility, and potential for field-deployable environmental diagnostics [149].

**Figure 8:**
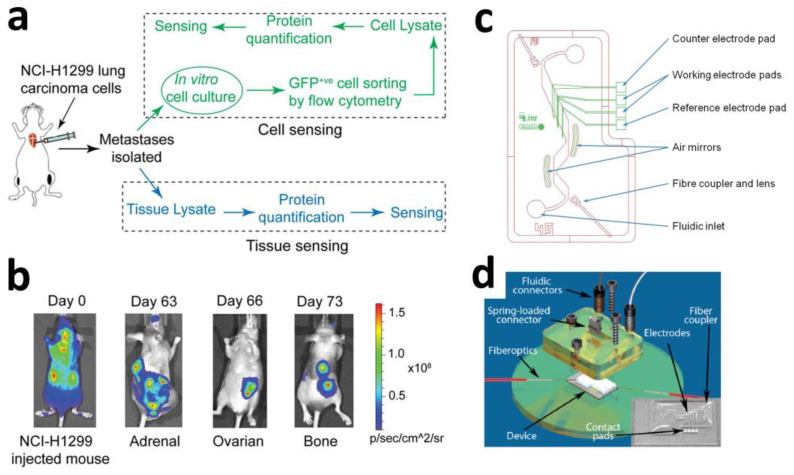
(**a**) Schematic presentation of generating site-specific metastatic cells, and tissues and their sensing using the selective array-based sensing approach. (**b**) Representative bioluminescence images of mice after intracardiac injection of EGFP-luc2-expressing NCI-H1299 cells (Day 0) and at different days showing adrenal, ovarian, and bone metastases [150]. Copyright © 2012, American Chemical Society. (**c**) Layout of the disposable DLOC. (**d**) Three-dimensional drawing of the DLOC interface assembly and a picture of the actual microdevice. Photonic elements, electrodes, and channels can be observed [154]. Copyright © 2012, American Chemical Society.

In cancer diagnosis and treatment, rapid and sensitive discrimination between metastatic and normal tissues is crucial for developing effective strategies. Subinoy Rana et al. developed an array based on gold-nanoparticle-coupled green fluorescent protein (GFP) sensing elements. This system utilizes the bright fluorescence signal of GFP for rapid visualization (response time within minutes), as shown in Figure 8a,b, enabling sensitive identification of metastatic cancer cells. More importantly, it creates a visualized “optical fingerprint” by integrating multiple fluorescence signal patterns (overall proteomic responses), thereby clearly distinguishing organ-specific metastases from their normal counterparts. Validated in a highly realistic preclinical metastasis model, this platform achieves precise discrimination with only minimal sample amounts (200 ng protein, ≈1000 cells), demonstrating the exceptional sensitivity of optically visualized multiplexed detection. The study includes both in vitro and in vivo validation: cultured parental and metastasis-derived sublines (adrenal, ovary, bone) were distinguished using lysate fluorescence profiles, while in vivo, organ-specific metastases were generated in a xenograft mouse model and compared directly with corresponding normal tissues. The sensor achieved 100% classification accuracy among tumor types and complete separation between malignant and healthy tissue fingerprints using linear discriminant analysis (LDA), demonstrating its robustness in handling tissue heterogeneity and translational applicability to clinical biopsy settings. Notably, this array approach provides an intuitive fluorescent visual “fingerprint” for discerning tissue states without relying on any predefined biomarker knowledge. This optically and nanoparticle-based visual sensing technology offers a powerful tool for rapid identification of cell/tissue states and exhibits versatility for constructing broad-spectrum detection platforms [150].

Single quantitative methods for serum H_2_S detection (a key biomarker for acute pancreatitis, AP) suffer from insufficient precision (high false-positive/negative rates). To overcome this limitation, Yuxin Liu et al. developed an innovative three-channel sensing platform (equipped with a dedicated instrument) capable of simultaneously implementing three distinct detection principles: electrochemical analysis, luminescence-based (optical) sensing, and photothermal-effect-based (thermal) sensing. The platform exploits a specific reaction with H_2_S, demonstrating excellent tri-modal (electrochemical–optical–thermal) performance within physiologically relevant concentration ranges. It effectively differentiated plasma H_2_S levels between AP model mice and normal mice. Particularly noteworthy, by integrating information from the electrochemical channel, the optical (luminescence) channel, and the thermal sensing (photothermal) channel and optimizing the diagnostic threshold, the precision of AP serodiagnosis was significantly improved (>99.0%), markedly surpassing that of strategies using a single channel (~79.5%) or dual channels (~94.1%) [151].

#### 2.4.2. Innovations in the Design and Fabrication of Multimodal/Signal Sensors

Based on the multi-signal sensing principles above, this section focuses on how micro/nano-fabrication and miniaturization technologies enable fully integrated, multi-channel diagnostic devices suitable for point-of-care applications.

The development of multimodal/signal biosensors is highly dependent on miniaturization and micro/nano-fabrication technologies. This is inherently a multidisciplinary process involving microfabrication, materials science, chemistry, biology, and engineering. Miniaturization technologies offer numerous advantages for the development of diagnostic devices: low manufacturing costs, minimal sample requirements, shortened reaction times, and the capability to perform different types of tests simultaneously on the same device [152]. Micro/nano-fabrication serves as the core manufacturing approach. Utilizing advanced techniques such as photolithography, thin film deposition, etching, microfluidics, nanomaterial integration, nanofabrication, and additive manufacturing, it constructs microstructures on-chip and enables the integration of multimodal signals. Equally crucial, microfabrication and microfluidic technologies are essential for constructing fully integrated electrochemical biodetection platforms aimed at the point-of-care (POC) market, possessing significant commercialization potential [153].

Olga Ordeig innovatively proposed a dual-mode integrated microfluidic detection system (DLOC), as shown in Figure 8c,d. Through a unique microfluidic chip design, it achieved, for the first time, the simultaneous operation of optical and electrochemical detection within a single detection volume. The core innovation of this system lies in its dual-function microfluidic chamber design, which employs precise fluidic control to ensure the absence of signal cross-talk between the two detection modes at a flow rate of 200 μL min^−1^. Furthermore, it constructs a multiparameter synchronous monitoring platform capable of real-time tracking of reactant consumption (electrochemical mode) and product generation (optical mode). In performance validation, the electrochemical detection of ferrocyanide (0.005–2 mM) achieved a sensitivity of 2.059 μA mM^−1^ (LOD 2.303 × 10^−3^ mM), while the optical detection of ferricyanide (0.005–0.3 mM) conformed to the Beer–Lambert law (LOD 0.553 × 10^−3^ mM). In the L-lactate detection application, the system realized the synchronous quantification of reactant (amperometry) and product (absorbance) through a bi-enzymatic LOX/HRP reaction, demonstrating not only sensitivity superior to conventional fluidic systems but also possessing a unique self-verifying capability. This multimodal integrated sensor design provides a new paradigm for microfluidic detection technology. Its ability to simultaneously acquire multidimensional information significantly enhances analytical reliability and accuracy [154].

An Ion-Sensitive Field-Effect Transistor (ISFET) is a representative electrochemical microsensor device. Regarding ISFET-based devices, Yamada et al. fabricated a microfluidic device for pH measurement [155]. Two ISFETs and one Ag/AgCl pseudo-reference electrode were employed for differential measurements. Sharma et al. fabricated a microfluidic chip with ISFETs for measuring pH and potassium ions.

## 3. Clinical and Health Management Applications

### 3.1. Dynamic Monitoring of Diseases

Dynamic monitoring of diseases constitutes a vital component of modern healthcare systems. It provides a scientific foundation for precise disease diagnosis and personalized therapeutic interventions by enabling continuous, real-time, or near-real-time data acquisition and analysis. With the evolving global disease spectrum and accelerating population aging, chronic non-communicable diseases such as neurological disorders, metabolic diseases, and cancer have emerged as major threats to human health. This section will delve into the importance of dynamic monitoring for these three categories of diseases, with a particular emphasis on the critical role of advanced functional nanomaterials within bioelectrode interfaces for disease detection and metabolic monitoring.

#### 3.1.1. Non-Metallic Materials Based Sensors

Monitoring neurological diseases presents unique challenges, dictated by the complexity of the nervous system and the diversity of disease manifestations. Common neurological disorders include Alzheimer’s disease, Parkinson’s disease, epilepsy, and stroke, which are often characterized by progressive and irreversible deterioration. Dynamic monitoring techniques are indispensable for timely detection of clinical changes, assessing disease progression, and evaluating treatment efficacy. For instance, in patients with acute brain injury, numerous non-convulsive seizures (NCSs) are occult, especially in those with impaired consciousness. These seizures are clinically unrecognizable yet cause secondary brain damage. Long-term continuous EEG monitoring (exceeding 24 h) is required to identify patients at risk of delayed seizures, thereby determining which patients require extended monitoring for accurate diagnosis, avoiding missed diagnoses and treatment delays [156].

Two-dimensional novel MXene nanomaterials composed of metal carbides or nitrides have been utilized to develop anti-artifact transparent microelectrode arrays. Sneha Shankar et al. fabricated a transparent microelectrode array based on Ti_3_C_2_T_x_ films, optimized for multimodal imaging and recording applications, enabling multimodal precision monitoring during epileptic seizures. Its characteristic 60% visible-light transmittance (at 550 nm) and low interfacial impedance (563 ± 99 kΩ at 1 kHz) effectively mitigate light-induced artifacts encountered in traditional electrodes during concurrent electrophysiological recording and optical imaging. Within an epilepsy model, this array successfully captured the electrographic signals during chemically induced seizures alongside the dynamic wavefront propagation of calcium imaging synchronously. Concurrently, it demonstrated the capability to capture high-frequency neuronal activity in rat barrel cortex across different cortical layers. Such a nanomaterial-driven platform with high spatiotemporal resolution provides integrated solutions for epilepsy research, combining neuronal activity recording, optical stimulation intervention, and real-time imaging monitoring.

**Figure 9:**
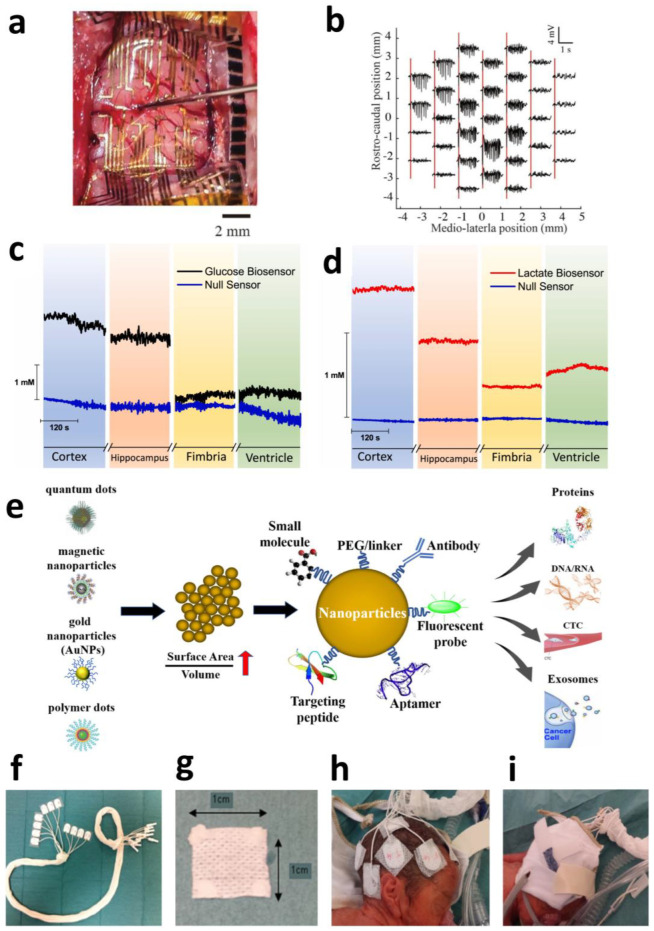
(**a**) Photograph of the electrode placed over the rat brain while bicuculline was injected via a Hamilton syringe to induce epileptiform activity. (**b**) Recording of pharmacologically induced epileptiform activity by the electrode. Black lines depict ECoG potentials from each channel, and the red line indicates the time point for generating the subsequent isopotential map [157]. Copyright © 2023, the author(s). (**c**,**d**) In vivo monitoring of glucose and lactate in four brain regions of Wistar rats: cortex, hippocampus, fimbria (white matter), and lateral ventricle fluid [161]. Copyright © 2021, Elsevier. (**e**) Nanotechnology improves cancer detection and diagnosis [192] Copyright © 2019, the author(s). (**f**–**i**) Key steps of EEG Application (**f**) Prelabeled electrodes positioned inside the stockinette. (**g**) Electrode with tape and paste. (**h**) Electrode positions on right hemisphere covered with tape. (**i**) Hat closed ready for recording [216]. Copyright © 2014, the author(s).

To address the issue of mechanical mismatch between traditional electrocorticography (ECoG) electrodes and the surface curvature of the cerebral cortex, which hinders long-term stable recordings, Ayano Imai et al. developed an ultra-flexible thin-film ECoG electrode (thickness ≈ 8 μm). This electrode employs a polystyrene–polybutadiene block copolymer (SBS) nanomembrane with excellent elasticity as both substrate and encapsulation layer, while integrating gold nanomaterial ink through inkjet printing technology to form a highly biocompatible conductive network. This composite nanomaterial structure significantly enhances the mechanical compatibility between the electrode and cortical tissue, supplemented by laser micromachining to optimize signal coupling via interfacial micro-nanostructures. Implanted on the rat cortical surface for up to 6 weeks, the device elicited no significant inflammation, confirming its exceptional biocompatibility and stability under long-term implantation scenarios. Leveraging high-density (35-channel) ECoG recording capabilities in epilepsy management, this electrode successfully achieved precise visualization of epileptiform activities in pharmacologically induced rat epilepsy models (Figure 9a,b). Concurrently, its integrated electrical stimulation functionality provides an advanced technical platform for exploring long-term closed-loop modulation strategies for drug-resistant epilepsy [157].

Following traumatic brain injury (TBI) and cerebral hypoxia, the brain frequently undergoes sustained energy metabolic crises and neurotransmitter dysregulation. These constitute core pathological mechanisms leading to secondary neuronal damage and poor outcomes. The imbalance in energy metabolites—characterized by acute glucose depletion and abnormal lactate accumulation—can persist from days to weeks [158], directly reflecting the severity and progression of impaired energy supply to neural tissue. Additionally, sustained abnormal release of excitatory neurotransmitters such as glutamate exacerbates excitotoxic injury. Long-term dynamic monitoring of these critical neurochemical markers is essential, enabling continuous tracking of pathological evolution and therapeutic efficacy. Substantial research has focused on utilizing diverse sensing technologies to detect specific cerebral neurochemicals for clinical diagnosis and neuroscience research [159,160].

Another study investigated post-treatment cerebral glucose changes. Matias Regiart et al., utilizing a glucose biosensor modified with nanoporous gold-platinum nanoparticles (NPG-PtNPs), achieved broad-range (0.2–200 μM) and highly selective dynamic monitoring of intracerebral glucose. This was enabled through synergistic interfacial catalytic enhancement (sensitivity: 5.96 A·M^−1^ cm^−2^) and anti-interference coating design. The nano-enzyme composite interface successfully supported long-term tracking of glucose metabolism across multiple rat brain regions. In developing sensors for cerebral neurochemical monitoring, many groups advocate for the use of multiple biomarkers to reveal reliable and comprehensive patterns of energy metabolism and neurotransmitter degradation. This study also demonstrated that the microbiosensor could be applied for the simultaneous determination of lactate and glucose concentrations (Figure 9c,d) in serum samples, facilitating in vivo measurements of these analytes in four distinct rat brain structures [161].

#### 3.1.2. Metabolic Disease Monitoring

Metabolic diseases have become a major challenge in global public health, with the incidence of diabetes, obesity, and metabolic syndrome continuing to rise. These diseases are characterized by an insidious onset, prolonged course, and frequent association with severe complications, imposing a heavy burden on both patients’ quality of life and healthcare systems. Epidemiological studies have demonstrated that metabolic diseases exhibit marked regional and population disparities. East Asian populations, owing to genetic predispositions (such as ALDH2 gene variants) and distinctive patterns of visceral fat distribution, are predisposed to these diseases even at relatively low body mass index (BMI) levels; conversely, in developing countries, incidence rates are escalating rapidly in tandem with urbanization [162].

Continuous glucose monitoring (CGM) measures interstitial fluid (ISF) glucose and provides information on continuous glycemic fluctuations that intermittent blood glucose testing cannot capture [163]. The application of CGM has transformed diabetes care from a reactive process into a proactive one, enabling patients to prevent hypoglycemic or hyperglycemic episodes rather than merely responding to them, and supporting effective intensive therapy to reduce glucose exposure and lower the risk of complications and hospitalization [164].

Fang et al. developed a flexible, needle-type microelectrode glucose biosensor modified with copper nanoflowers for continuous in vivo glucose monitoring. In this study, copper nanoflowers with a high specific surface area and electrocatalytic activity were fabricated via a one-step electrodeposition method, and a PU membrane was employed as a mass-transport limiting layer, significantly enhancing the sensor’s linear range and stability. The developed biosensor exhibited a good linear response over the 0–20 mM glucose concentration range (sensitivity 42.38 nA mM^−1^) with a response time of less than 15 s. In anesthetized rat in vivo experiments, the sensor was able to respond to changes in blood glucose concentration in real-time, and its output trend was consistent with that of a conventional glucometer, providing a promising implantable monitoring solution for medical diagnostic applications such as diabetes management [165].

Liu et al. developed a high-sensitivity, wearable field-effect transistor (FET) biosensor based on In_2_O_3_ nanobelts for glucose monitoring in body fluids. By integrating an on-chip gold side-gate design, this study addressed the reliance of traditional FET biosensors on bulky Ag/AgCl electrodes, enabling conformal application to surfaces such as artificial arms and wristwatches. The device, fabricated via a shadow-mask method, exhibited an electron mobility of approximately 22 cm^2^ V^−1^ s^−1^ and an on/off ratio of 10^5^ in 0.1× PBS, along with excellent mechanical stability. Through functionalization with glucose oxidase, chitosan, and single-walled carbon nanotubes, the sensor achieved a broad detection range spanning five orders of magnitude and a detection limit as low as 10 nM, providing a key component for wearable medical electronic devices [166].

Tawakey et al. developed a biosensor based on a microneedle electrode array for ultrasensitive monitoring of glucose in interstitial fluid, enabling early detection of blood glucose abnormalities. In this study, polymethyl methacrylate (PMMA) microneedle glucose sensors were fabricated using 3D printing, microfabrication, and electrodeposition techniques, and glucose detection was achieved through an enzyme immobilization step. In vitro testing demonstrated that the sensor exhibited a linear response over the 1.5–14 mM glucose concentration range (sensitivity 1.51 μA mM^−1^), with a detection limit of 0.35 mM, and showed high selectivity against physiological interferents such as ascorbic acid, uric acid, and mannose. The integrated system is also equipped with an emergency alarm function capable of sending alerts via email or SMS upon detection of abnormal glucose values, providing continuous monitoring and timely warnings for diabetic patients [4].

Zhang et al. developed an ultrasensitive, label-free surface-enhanced Raman scattering (SERS) nanosheet based on physically etched silver films for the specific detection of glucose in body fluids. In this study, a physical scratching method was employed to fabricate an AgNS600 substrate featuring nanoscale groove architectures that effectively trap glucose molecules and generate multidimensional “hotspot” regions, achieving an exceptionally low detection limit of 0.5 amol L^−1^. The platform was further integrated with machine learning algorithms capable of discriminating among various monosaccharides, polysaccharides, and their mixtures, and successfully detected glucose signals in real samples including blood, urine, tears, and sweat [167].

Liu et al. developed a wearable, rapid-fabrication, and stability-enhanced microneedle patch for closed-loop diabetes management. In this study, sensors printed with graphene-composite ink were integrated with hollow microneedles, and a polyethylene glycol (PEG)-functionalized electroosmotic micropump was incorporated to enable interstitial fluid glucose sensing and intelligent insulin delivery control. PEG functionalization markedly improved the stability of insulin delivery, extending the micropump’s operational lifespan from several days to several weeks. In diabetic rat models, the microneedle patch demonstrated excellent glycemic control, offering a novel design paradigm for closed-loop diabetes management systems [168].

Anuthum et al. developed a dual-mode electrochemical biosensor based on a gold nanoparticle/2D MoWS_2_/graphene oxide nanocomposite for the simultaneous detection of glycated albumin (GA) and glucose. In this study, polyethyleneimine-modified gold nanoparticles, graphene oxide, and a two-dimensional molybdenum–tungsten disulfide composite were integrated onto a screen-printed carbon electrode to construct a dual-working-area sensor combining competitive immunoassay and enzymatic detection capabilities. The biosensor demonstrated excellent performance in 50-fold diluted human serum, with a GA detection range of 500–25,000 pg mL^−1^ (limit of detection 320 pg mL^−1^) and a glucose detection range of 0.5–8.0 mM (limit of detection 0.15 mM). This dual-mode design, which integrates both short-term and mid-term glycemic markers, significantly enhances the accuracy of diabetes diagnosis and offers a novel strategy for the development of more comprehensive diabetes-monitoring systems [169].

Carbon nanomaterials (e.g., carbon nanotubes, graphene, carbon quantum dots) are widely used in electrochemical sensors owing to their excellent electrical conductivity and high surface activity. These materials enhance the catalytic activity of electrode surfaces and improve signal response. For example, graphene-based nanocomposites can serve as electrode substrates for the electrochemical oxidation or reduction detection of metabolites such as glucose, lactate, and cholesterol [170,171]. Prussian blue/nitrogen-doped carbon nanotube (PB/N-doped CNT) composites exploit a molecular-structure matching mechanism to achieve specific electrochemical detection of uric acid (linear range of 0.1–150 U/L), facilitating gout monitoring. In addition, nanozymes (e.g., Fe_2_O_3_ and MnO_2_ nanomaterials) mimic the catalytic activity of natural enzymes by catalyzing hydrogen peroxide (H_2_O_2_) to generate free radicals, thereby enabling the detection of various metabolic analytes.

Nanomaterials have been extensively applied in optical sensing, particularly leveraging fluorescence and surface plasmon resonance (SPR) effects. For instance, quantum dots (QDs) and graphene quantum dots, owing to their high fluorescence quantum yields and tunable optical properties, have been employed for the detection of metabolites such as glucose, uric acid, and vitamin [172]. In addition, metal nanoparticles (e.g., gold and silver nanoparticles) exploit SPR effects by monitoring changes in their absorption spectra to quantify metabolites. For example, gold nanoparticles undergo biomolecule-induced self-assembly upon binding to analytes such as uric acid [173], resulting in measurable shifts in their UV–visible absorption spectra for quantitative analysis. MnO_2_ nanosheets, in conjunction with fluorescent PDA, employ a FRET mechanism to achieve ultrasensitive detection of alkaline phosphatase (ALP) activity (limit of detection of 0.15 mU/mL), thereby facilitating the monitoring of hepatic and skeletal metabolic disorders [174].

Nanothermodynamic sensors utilize thermopile technology to monitor metabolites by detecting heat exchange between the sample and nanomaterials. For example, a portable biosensor based on nanothermodynamic principles employs calorimetry to measure hydrogen peroxide (H_2_O_2_) and phenylalanine (Phe) in fingertip blood, enabling real-time monitoring of metabolic diseases such as diabetes and hyperlipidemia. Calorimetric detection does not require secondary or labeled reactions for signal transduction, thus offering directness and high sensitivity [175].

Nanomaterials can also achieve specific recognition of metabolites through conjugation with biomolecules such as antibodies, enzymes, or DNA. For instance, graphene-based nanocage dual-enzyme complexes mimic the cooperative action of endogenous enzymes to enhance catalytic efficiency. Surface functionalization of nanomaterials, for example, by introducing ligands, antibodies, or DNA, further improves specificity toward target metabolites. Moreover, the high specific surface area and porous architecture of nanomaterials enable efficient signal amplification, thereby increasing detection sensitivity [176].

#### 3.1.3. Cancer Biomarker Monitoring

Cancer is the second leading cause of death worldwide, and its early detection and precision therapy remain paramount objectives in clinical medicine. Compared with conventional imaging techniques, dynamic monitoring of tumor biomarkers provides molecular-level information, enabling the detection of abnormalities before anatomical changes occur, and thus offering unique value for cancer diagnosis, therapeutic efficacy evaluation, and relapse surveillance [177]. With advances in detection technologies and the discovery of novel biomarkers, cancer biomarker monitoring is evolving toward greater precision and dynamism.

Molecular tumor biomarkers and nanomaterial-based biosensors offer significant advantages for early cancer screening and diagnosis. Compared with conventional imaging modalities, these approaches enable highly sensitive detection at the molecular level in the very earliest stages of tumor development, substantially advancing the screening window for high-risk populations.

Chang et al. developed a homogeneous electrochemical biosensor based on nucleic-acid-functionalized MOFs for the simultaneous detection of multiple tumor biomarkers. In this study, UIO-66-NH_2_ served as a nanoscale container to load electroactive dyes (MB and TMB), and a double-stranded DNA (dsDNA) gating mechanism was employed to achieve specific recognition of let-7a and miRNA-21. In the presence of the target miRNAs, hybridization with the probe DNA formed duplexes that triggered the release of the electroactive dyes, generating concentration-dependent electrochemical signals with detection limits of 3.6 fM for let-7a and 8.2 fM for miRNA-21 [178].

Li et al. developed an ultrasensitive biosensor based on a silicon nanowire-array field-effect transistor (SiNW-array FET) for the quantitative detection of circulating tumor DNA (ctDNA). In this study, CMOS-compatible microfabrication techniques were used to fabricate a highly responsive array of 120 silicon nanowires on a silicon-on-insulator (SOI) substrate, and DNA probes were immobilized onto the nanowire surfaces via silanization. The experimental results demonstrated a limit of detection for PIK3CA E542K ctDNA of 10 aM (10^−18^ M), with a linear response over the 0.1 fM to 100 pM concentration range, and the capability to distinguish fully matched DNA sequences from single- and double-base mismatches [179].

Xu et al. developed a self-powered biosensor platform based on a novel sandwich-structured graphdiyne (SGDY) for ultrasensitive detection of tumor biomarkers. In this study, the SGDY material was combined with aptamer-specific recognition functionality to achieve high-sensitivity detection of miRNA-21 and miRNA-141, with detection limits of 0.15 fM and 0.30 fM, respectively. The sensor exhibited a linear response over the ranges of 0.05–10,000 fM for miRNA-21 and 1–10,000 fM for miRNA-141 [180].

Wang et al. developed a SPR biosensor based on antibody–quantum dot conjugate signal amplification for the quantitative detection of multiple tumor biomarkers. In this study, a dual signal amplification strategy employing gold nanoparticle–antibody conjugates and antibody–quantum dot conjugates enhanced detection sensitivity by 50-fold, enabling ultrasensitive detection of alpha-fetoprotein (AFP), carcinoembryonic antigen (CEA), and cytokeratin fragment 21-1 (CYFRA 21-1), with a limit of detection down to 0.1 ng/mL. The experimental results demonstrated that the SPR biosensor’s measurements showed good concordance with those obtained by electrochemiluminescence assays [181].

Xu et al. first developed an aptamer-induced “switchable” fluorescence resonance energy transfer (FRET) biosensor capable of dual-color, synchronous detection of multiple tumor biomarkers under single-wavelength excitation. In this study, MoS_2_ nanosheets were combined with multicolor gold nanoclusters (Au NCs), employing green Au NCs (510 nm) functionalized with an AFP aptamer and red Au NCs (650 nm) functionalized with a CEA aptamer as energy donors, with MoS_2_ serving as the energy acceptor. This configuration enabled simultaneous detection of AFP and CEA, achieving limits of detection of 0.16 ng/mL and 0.21 ng/mL, respectively. Confocal microscopy experiments confirmed the sensor’s ability to distinguish between serum samples from healthy individuals and hepatocellular carcinoma patients, demonstrating promising clinical diagnostic potential [182].

Shi et al. provided a comprehensive review of signal amplification strategies in photoelectrochemical (PEC) biosensors for tumor marker detection over the past five years. The authors highlighted that newly developed PEC sensors, owing to their high sensitivity and low cost, have emerged as one of the most promising methods in the field of tumor marker analysis. They emphasized that the integration of PEC technology with biosensing strategies offers superior specificity and sensitivity compared with traditional detection methods [183].

Cheung et al. systematically reviewed the clinical utility of tumor biomarkers in the diagnosis and therapeutic monitoring of breast cancer. The study noted that elevated blood levels of tumor markers correlate with breast cancer staging and that the combined measurement of CEA and mucin-1 (CA15-3 or CA27.29) is of significant value in diagnosing metastatic breast cancer. For monitoring treatment response in advanced breast cancer, the combination of CA15-3, CEA, and ESR demonstrated good concordance with the conventional UICC criteria, while offering operational simplicity [177].

Molecular tumor biomarkers coupled with nanomaterial-based biosensors have markedly enhanced the sensitivity and efficiency of early cancer screening, and hold promise as critical tools for early warning and precise diagnosis in high-risk populations.

Cancer biomarker monitoring is becoming increasingly precise and dynamic owing to the application of high-sensitivity detection techniques, such as droplet digital PCR (ddPCR) and novel biosensors. Droplet digital PCR and nanomaterial-based biosensors substantially enhance the sensitivity, specificity, and quantitative capabilities of cancer biomarker detection, thereby facilitating early diagnosis, therapeutic monitoring, and the detection of minimal residual disease (MRD).

Postel et al. systematically reviewed circulating tumor DNA (ctDNA) detection methods based on ddPCR and optimized next-generation sequencing (NGS) technologies in cancer diagnosis. The study highlighted that these emerging techniques markedly improve the sensitivity, specificity, and accuracy of trace ctDNA detection in blood, offering new avenues for relapse surveillance, MRD detection, and early diagnosis. Compared with traditional tissue biopsy, ctDNA assays provide minimally invasive sampling, high reproducibility, and a more comprehensive reflection of tumor heterogeneity and multifocal disease characteristics [184].

Mangolini et al. employed droplet digital PCR (ddPCR) to quantitatively analyze microRNAs (miRNAs) in the serum of breast cancer patients. In two independent cohorts, the study found that serum levels of miR-148b-3p and miR-652-3p were significantly lower in breast cancer patients than in healthy controls, whereas elevated miR-10b-5p expression was associated with poor prognosis. Receiver operating characteristic analysis confirmed the diagnostic value of these miRNAs for distinguishing breast cancer patients from healthy individuals [185].

Huang et al. developed an ultrasensitive biosensor based on droplet digital branched rolling circle amplification (ddBRCA) for the detection of exosomes derived from gastric cancer cells. By designing a hairpin-structured DNA containing both an exosome-specific aptamer sequence and BRCA primer sequences, the platform enables target-triggered signal amplification. The assay achieved a detection limit as low as 12 particles/μL. Encapsulating the reaction mixture within nanoliter-scale droplets and incubating at 30 °C for 45 min, the entire workflow is completed in approximately 2 h without requiring wash steps [186].

High-sensitivity droplet digital PCR and novel nanomaterial-based biosensors are driving cancer biomarker monitoring toward greater precision, dynamism, and diversity. These technologies markedly enhance capabilities for early diagnosis, therapeutic efficacy evaluation, and minimal residual disease detection, providing powerful tools for personalized management and follow-up of cancer patients.

The application of multiplex biomarker detection and new biosensors based on nanomaterials significantly elevates management capabilities and detection sensitivity throughout the entire cancer care continuum. Cancer biomarker monitoring is evolving toward multiplexed, dynamic, and ultra-sensitive platforms; nanomaterials and advanced sensor technologies greatly improve detection efficiency and accuracy, offering robust support for comprehensive cancer management and individualized therapy.

Cheung et al. systematically evaluated the clinical utility of tumor biomarkers in breast cancer diagnosis and therapeutic monitoring. The study indicated that combined measurement of blood tumor biomarkers—including CEA, mucin-1 antigens (CA15-3 or CA27.29), and erythrocyte sedimentation rate (ESR)—provides significant value for diagnosing metastatic breast cancer and monitoring treatment response, outperforming single-marker assays and demonstrating strong concordance with conventional UICC criteria. Although the use of tumor biomarkers for early screening of primary breast cancer requires further validation, their application in monitoring metastatic lesions has already shown considerable clinical benefits [177].

Wilson et al. developed an immunomagnetic capture and multicolor surface-enhanced Raman scattering (SERS) nanolabel-based method for multiplexed detection of surface markers on circulating tumor cells (CTCs). In this study, magneto-plasmonic iron-oxide–gold core–shell nanoparticles were used to fabricate four-color SERS nanolabels, which, in combination with a microfluidic chip, enabled the simultaneous quantification of four surface protein markers on single tumor cells in whole blood samples. The experimental results demonstrated a strong correlation between SERS-based measurements and enzyme-linked immunosorbent assay (ELISA) for breast cancer cell-line surface markers, with significantly enhanced detection sensitivity. This integrated platform, combining immunomagnetic enrichment, multiplexed targeted capture, and high-sensitivity SERS detection, provides a novel tool for the sensitive monitoring of cancer metastasis [187].

Xu et al. developed a dual-color DNA–silver nanocluster (AgNC) fluorescent aptamer sensor based on salt-induced gold nanoparticle aggregation for the simultaneous detection of CEA and cancer antigen 125 (CA125). In this approach, green-emitting DNA-AgNCs functionalized with a CEA aptamer (gDNA1-AgNCs-apta1), and red-emitting DNA-AgNCs functionalized with a CA125 aptamer (rDNA2-AgNCs-apta2) were mixed in a 1:1 volume ratio to construct a dual-color detection system. Upon target binding, aptamer-AgNC complexes dissociate from the gold nanoparticle surface, inducing salt-triggered gold nanoparticle aggregation and consequent fluorescence recovery. The method was successfully applied to the selective simultaneous detection of CEA and CA125 in human serum samples, offering a new high-sensitivity platform for the clinical diagnosis of ovarian cancer [188].

Cancer biomarker monitoring is undergoing a rapid transformation, driven by the integration of emerging technologies such as nanomaterial-based biosensors, liquid biopsy, and artificial intelligence. These advances have significantly enhanced the precision, efficiency, and dynamic nature of cancer detection, and facilitated the development of non-invasive and real-time precision medicine.

Amor et al. developed an artificial-intelligence-based nanoarray sensor for liquid biopsy diagnosis of multiple cancers by detecting volatile organic compound (VOC) patterns in blood headspace. The study utilized a sensor system composed of chemically sensitive nanostructured thin films to detect and stage three prevalent cancers—breast, ovarian, and pancreatic cancer. The system demonstrated >84% accuracy in early-stage cancer detection and exceeded 97% accuracy in metastatic cancer diagnosis. Mass spectrometry analysis validated the reliability of the method, confirming its ability to simultaneously analyze diverse VOC datasets and significantly enhance the sensitivity of the predictive model [189].

Jopek et al. developed a deep-learning-based multi-class cancer classification algorithm that enables liquid biopsy diagnosis through analysis of RNA expression profiles in tumor-educated platelets (TEPs). The study used KEGG pathway gene expression heatmaps as input to train an optimized ResNet9-implant model, which achieved a balanced accuracy of 66.51% in distinguishing six cancer types, with accuracy increasing to 90.5% in location-specific subgroup datasets. By incorporating explainable artificial intelligence (XAI) techniques such as SHAP, the study identified 60 key genes most influential in model decision-making, including known cancer biomarkers such as DIAPH1 and ACER2. This integration of liquid biopsy and deep learning offers a novel solution for clinical early cancer detection and classification [190].

Nanomaterials can enhance the sensitivity and specificity of cancer biomarker detection through their high specific surface area and pronounced surface effects. Their large surface-to-volume ratio allows for the loading of multiple targeting molecules, thereby amplifying detection signals. For example, gold nanoparticles (AuNPs) and QDs, owing to their high surface area, can be functionalized with a variety of biomolecules—such as antibodies, nucleic acid aptamers, and small molecules—to achieve specific recognition of cancer cells and tissues [191]. Furthermore, surface functionalization of nanomaterials with peptides, antibodies, aptamers, or small molecules can further augment their targeting capability and detection performance [192].

Surface effects endow nanomaterials with exceptional electronic, magnetic, mechanical, and optical properties when integrated into biosensing platforms. For instance, graphene oxide (GO) modified with aptamers has achieved a detection limit for CEA as low as 0.3 pg/mL, outperforming ELISA by three orders of magnitude [193]. In addition, synergistic effects between conductive polymers and noble-metal nanomaterials have been exploited to significantly enhance the sensitivity and specificity of electrochemical immunosensors [194].

The integration of nanomaterial-based biosensors, liquid biopsy, and artificial intelligence is propelling cancer biomarker monitoring toward greater precision, dynamism, non-invasiveness, and individualization. Advances in these technologies hold great promise for significantly enhancing the efficiency and accuracy of early cancer screening, tumor classification, and therapeutic monitoring, thereby providing a solid foundation for precision medicine.

### 3.2. Nanomaterial-Driven Early Diagnostic Innovations

Advancements in early diagnostic technologies are reshaping modern healthcare systems, offering new possibilities for disease prevention and control. From tumor diagnostics to the detection of biomarkers for neurodegenerative diseases, from pediatric electroencephalographic monitoring to the analysis of developmental metabolic indicators, technological innovations are breaking through the limitations of traditional medicine. Functional nanomaterials for advanced bioelectrode interfaces play a pivotal role in enabling high-precision and dynamic monitoring. The development of these technologies has significantly improved the accuracy and efficiency of diagnostic procedures.

#### 3.2.1. Nanomaterial-Enhanced Early Tumor Diagnostics

Significant breakthroughs have been achieved in the field of early tumor diagnosis in recent years, with the emergence of various innovative technologies substantially enhancing the sensitivity and specificity of cancer screening. Liquid biopsy technology, one of the most representative advancements, enables early detection of malignant tumors such as hepatocellular carcinoma by analyzing circulating tumor DNA (ctDNA) and specific protein biomarkers in the blood. A technology jointly developed by the Cancer Hospital of the Chinese Academy of Medical Sciences and Genetron Health allows for early-stage liver cancer screening from asymptomatic hepatitis B virus carriers with a single blood draw. This method has undergone rigorous clinical validation, offering a promising pathway for establishing a convenient, non-invasive, and standardized early screening protocol for liver cancer.

Nanomaterials are propelling the development of integrated tumor theranostics. In tumor detection and sensing, nanomaterials not only improve the detection limit but also enhance accuracy and enable in situ imaging approaches. For instance, gold nanorods (AuNRs) functionalized with anti-HER2 antibodies can differentiate malignant from non-malignant cells under dark-field microscopy with > 95% accuracy [195]. Magnetic nanoparticles (Fe_3_O_4_) have been utilized to isolate exosomes, which are then labeled with quantum dots to enable in situ imaging of lung cancer-associated miRNAs (Figure 9e) [192]. Multimodal imaging-guided therapy holds great promise in the application of nanomaterials for cancer diagnosis, incorporating modalities such as magnetic resonance imaging (MRI), fluorescence imaging, photoacoustic imaging, and positron emission tomography (PET). For example, superparamagnetic nanoparticles and quantum dots have been used to enhance imaging resolution and improve early cancer detection capabilities [196,197].

Overcoming multidrug resistance (MDR) is another key area where nanomaterials play a vital role in disease intervention. By modulating the tumor microenvironment—such as pH levels and hypoxia—and increasing intracellular drug concentrations, nanomaterials can overcome tumor MDR. Additionally, they can regulate autophagy and apoptosis pathways to enhance tumor cell sensitivity. Dendritic polymer-based nanocarriers can co-deliver chemotherapeutic agents and gene-silencing RNAs to reverse tumor resistance [198,199].

In terms of nanoprobe-based biomarker detection, nanomaterials are employed to detect circulating tumor cells (CTCs) and tumor biomarkers such as folate receptors, EGFR, and HER2, enabling early diagnosis and prognostic assessment [200].

#### 3.2.2. Advanced Biosensors for Neurodegenerative Disease Detection

Early diagnosis of neurodegenerative diseases has long posed a major challenge to the medical community. Recent breakthroughs in biomarker detection technologies have brought new hope to this field. A research team from École Polytechnique Fédérale de Lausanne (EPFL) in Switzerland developed the “ImmunoSEIRA” biosensor, representing a significant advancement in this domain. This sensor is capable of accurately detecting misfolded protein biomarkers associated with diseases such as Parkinson’s disease and Alzheimer’s disease [201]. This innovative technology integrates multiple disciplines, including protein biochemistry, optics, nanotechnology, and artificial intelligence. It utilizes surface-enhanced infrared absorption (SEIRA) spectroscopy, in combination with gold nanorod arrays and specific antibodies, to enable real-time capture and structural analysis of target biomarkers in ultra-trace samples.

The early diagnosis of neurodegenerative diseases, such as Alzheimer’s disease and Parkinson’s disease, has long faced significant challenges. In recent years, biosensor technologies based on nanomaterials have dramatically improved the sensitivity and accuracy of detecting disease-associated biomarkers, providing breakthrough advances for early, non-invasive, and efficient diagnosis.

Mars et al. developed a dual-mode sensing platform based on curcumin-functionalized graphene quantum dots for electrochemical and fluorescence detection of Alzheimer’s disease-related APOe4 DNA. In this study, curcumin molecules were electropolymerized on the surface of indium tin oxide (ITO) electrodes, and amine-modified DNA probes were covalently immobilized via EDC/NHS chemistry to construct a biosensor with dual detection functionality. This platform demonstrated excellent analytical performance, with an electrochemical detection sensitivity of 4.74 nA·mL·pg^−1^ and a detection limit as low as 0.48 pg·mL^−1^, along with good repeatability, selectivity, and long-term stability [202].

Azimzadeh et al. developed an ultrasensitive electrochemical biosensor based on electrochemically reduced graphene oxide (ERGO) and AuNWs for the quantitative detection of microRNA-137 (miR-137) in serum as a biomarker for Alzheimer’s disease (AD). The study involved the modification of screen-printed carbon electrodes (SPCEs) and the incorporation of the embedded marker doxorubicin (Dox) to construct a nanobiosensor with excellent analytical performance. The experimental results demonstrated a good linear response within the range of 5.0 to 750.0 fM, with a detection limit as low as 1.7 fM. The sensor effectively distinguished the target oligonucleotide from non-specific oligonucleotides, including single-base mismatches, triple-base mismatches, and non-specific miR-21 and miR-155 [203].

Lu et al. developed a voltametric detection technique based on ferrocene-modified gold nanoparticles for the direct detection of the Alzheimer’s disease-associated ApoE4 gene. In this study, streptavidin-modified ferrocene-gold nanoparticles were used as signal amplification elements, and the restriction endonuclease HhaI was employed for specific recognition of the GCGC sequence to achieve selective detection of the ApoE4 gene. The method exhibited a low detection limit of 0.1 pM and effectively differentiated the ApoE4 gene from other ApoE gene sequences [204].

Carneiro et al. developed a highly sensitive label-free electrochemical immunosensor for the detection of β-amyloid protein 1–42 (Aβ(1–42)), a major biomarker of Alzheimer’s disease (AD). The study constructed the immunosensor by modifying a gold electrode with a self-assembled monolayer of mercaptopropionic acid, electrodeposited gold nanoparticles, and a thiolated monoclonal antibody mAb DE2B4. The sensor demonstrated excellent analytical performance, exhibiting a good linear response in the range of 10–1000 pg mL^−1^, with a detection limit and quantification limit of 5.2 pg mL^−1^ and 17.4 pg mL^−1^, respectively. The recovery rate ranged from 90.3% to 93.6%. This low-cost, high-sensitivity immunosensor (14.6% signal decrease·mL·pg^−1^) offers a novel tool for in vitro diagnosis and in vivo investigation of Alzheimer’s disease [205].

Qin et al. developed a highly sensitive electrochemical impedance sensor based on cellular prion protein (PrPC) for the detection of β-amyloid oligomers (AβO). In this study, the PrPC bioreceptor was conjugated to a poly(3-thiopheneacetic acid) transducer, and a conductive layer composed of poly(3,4-ethylenedioxythiophene) embedded with gold nanoparticles was employed to fabricate a composite electrode with excellent conductivity and a high specific surface area. The sensor exhibited an ultralow detection limit at the sub-femtomolar level and a wide detection range from 10^−8^ to 10^4^ nM. Its applicability was validated in a mouse model of Alzheimer’s disease (AD) [206].

Bilal et al. systematically reviewed the advances in the application of nanomaterials for the diagnosis and treatment of Alzheimer’s disease (AD). The study highlighted that nanocarriers, such as magnetic nanoparticles, carbon nanotubes, quantum dots, and polymeric nanocapsules, exhibit unique advantages in the treatment of neurodegenerative diseases by enabling controlled drug delivery, surface functionalization, and the ability to cross the blood–brain barrier (BBB). In the diagnostic domain, biosensors based on nanomaterials such as graphene and gold/silver nanoparticles significantly enhance the sensitivity and selectivity for the detection of AD biomarkers [207].

Amor-Gutiérrez et al. developed a competitive electrochemical immunosensor based on gold nanoparticle-modified screen-printed carbon electrodes for the detection of a novel Alzheimer’s disease (AD) biomarker—unfolded p53 protein. The study utilized antibodies that specifically recognize epitopes of the unfolded p53 protein, achieving a wide dynamic range of 2–50 nM and a low detection limit of 0.05 nM. This sensor successfully quantified unfolded p53 protein levels in plasma samples from healthy elderly individuals, patients with mild cognitive impairment (MCI), and AD patients, showing no significant difference when compared to ELISA results [208].

Innovative approaches involving nanomaterials for the detection of biomarkers associated with neurodegenerative diseases encompass multiple aspects, including high-sensitivity detection, targeted delivery, imaging, and therapeutic interventions, offering new strategies and tools for the early diagnosis and treatment of such disorders.

Nanocarriers such as nanoliposomes, polymeric nanoparticles, and carbon nanotubes have been applied in the treatment of Alzheimer’s and Parkinson’s diseases. These systems can cross the BBB through surface functionalization, enabling targeted drug delivery and real-time monitoring of central nervous system biomarkers (e.g., β-amyloid protein), thereby improving drug bioavailability and reducing side effects [209,210].

Nanosensors, combining the high sensitivity of nanomaterials with biorecognition elements (e.g., antibodies, aptamers), allow for ultra-sensitive detection of neurodegenerative disease-related biomarkers. For instance, electrochemical sensors based on carbon nanomaterials and gold nanoparticles can detect picomolar levels of β-amyloid oligomers and tau protein. Electrodes modified with thionine/reduced graphene oxide/multi-walled carbon nanotubes (TH/rGO/MWCNTs) composites have achieved a detection limit as low as 0.01 pM. Aptamer sensors based on poly(thionine)/gold nanostars (pTH/Au NSs) have been developed for detecting α-synuclein (α-syn), with a remarkable detection limit of 0.03 fM, enabling early diagnosis of Parkinson’s disease.

The integration of nanomaterials with stem cells offers therapeutic potential for neurodegenerative disorders. Nanomaterials can act as carriers for stem cells, promoting the differentiation and migration of neural stem cells. For example, gold nanoparticles can enhance hippocampal neurogenesis under electromagnetic stimulation, while single-stranded DNA-functionalized gold nanoparticles can promote neuronal differentiation under circularly polarized light [211].

#### 3.2.3. Nanomaterial-Enhanced EEG Monitoring for Pediatrics

Significant advances have been made in pediatric EEG monitoring and the tracking of growth and developmental metabolic indicators in recent years, providing more precise tools for the diagnosis of pediatric diseases and the assessment of health. In the field of EEG monitoring, the cognitive function screening system developed by Zhongke Ruiyi enables home-based cognitive function evaluation and health management for children. This convenient monitoring method is particularly well suited for long-term tracking of neurological development in children. Compared with conventional EEG, modern high-density EEG (HD-EEG) and quantitative EEG (qEEG) technologies offer more accurate localization and quantitative analysis of EEG signals, providing an objective basis for the early identification of neurodevelopmental disorders in children, such as attention-deficit/hyperactivity disorder (ADHD) and autism spectrum disorder (ASD).

Continuous EEG monitoring (cEEG) is the most widely used neuro-monitoring modality in pediatric intensive care units and has become the gold standard for diagnosing seizures and assessing treatment responses in critically ill children. The analysis and visual trend mapping of qEEG extracts and quantifies features from the raw digital EEG, thereby allowing for the display of EEG characteristics and the time-compressed visualization of EEG data. This analytical approach enables the detection of subtle changes occurring over the course of several hours—changes that are not easily observed through frame-by-frame review of the raw EEG—significantly enhancing monitoring efficiency. In neonatal neuro-monitoring, a modified “bipolar montage” employing fewer electrodes has been adopted to accommodate the smaller head size of neonates while simultaneously reducing the risk of skin injury.

Current research on pediatric EEG monitoring combined with dry electrodes indicates that dry electrodes offer significant advantages in neonatal and pediatric EEG applications. Unlike wet electrodes, dry electrodes do not require conductive gel, and they feature portability, painlessness, and safety, while providing signal quality comparable to that of wet electrode systems [212]. Moreover, the design of dry electrodes (such as normal leg-shaped electrodes and flat disc electrodes) and the flexibility of electrode caps make them suitable for children of various age groups [213]. Demonstrating good biocompatibility, experimental results [214] have confirmed their non-cytotoxicity, rendering them suitable for the sensitive scalps of children. Semi-dry hydrogel electrodes (e.g., AgNW/PVA composites) can continuously release electrolytes [215], maintaining low impedance (6–8.5 kΩ) for up to 10 h and reducing discomfort during prolonged wear. In neonatal intensive care, dry electrodes have also been employed for continuous EEG monitoring to assess brain function and seizure activity (Figure 9f–i) [216]. However, in certain circumstances, dry electrodes may not fully substitute wet electrodes—for example, when high-precision signal recording or the detection of specific EEG activities is required. Therefore, despite their broad clinical application prospects, dry electrodes may still need to be complemented by wet electrodes or used in conjunction with multichannel monitoring in complex cases.

Pediatric electroencephalographic monitoring sensors can be enhanced using a variety of nanomaterials, particularly in the detection of disease-related signals, where these pediatric EEG sensors play a significant role.

#### 3.2.4. Nanomaterial-Based Sensors for Monitoring Growth and Metabolic Indicators

Bioelectrical impedance analysis (BIA) is a non-invasive measurement technique that assesses nutritional status by measuring the composition of tissues (such as fat, muscle, and water) in different parts of the body. The application of this technology in pediatric nutritional monitoring helps accurately determine whether a child’s growth and development are normal and whether issues such as malnutrition or obesity are present.

Medical imaging techniques such as ultrasound, X-ray, and MRI can be used to assess skeletal development and the condition of internal organs in children. These technologies provide intuitive visualization of the child’s internal structures, offering direct evidence for clinicians to assess nutritional status.

Growth hormone (GH) and gonadotropins (FSH, LH) are critical indicators for evaluating children’s growth and development. Monitoring these hormone levels enables the timely detection of endocrine disorders such as adrenal insufficiency or growth hormone deficiency.

In the monitoring of growth and metabolic indicators, the unique properties of nanomaterials (with sizes ranging from 1 to 100 nm) make them ideal tools for pediatric metabolic monitoring [217]. Graphene-based sensors can detect as few as 3–5 cancer cells/mL in blood and exhibit significantly enhanced precision when applied to the detection of trace pediatric metabolites such as IGF-1. Zero-dimensional quantum dots (0D) offer high sensitivity and precise fluorescent labeling; one-dimensional nanowires (1D) serve in constructing electrochemical sensors; two-dimensional graphene (2D) supports flexible wearable devices tailored to the active behavior of children [218].

In terms of biocompatibility and safety, optimized organic nanomaterials—such as liposomes and dendrimers—are biodegradable and exhibit low toxicity, making them suitable for long-term use in children. Surface modifications (e.g., polyethylene glycol coating) reduce immunogenicity [219].

In plant growth monitoring, the real-time tracking capabilities of nanomaterials have also been applied. Qi Zhang et al. developed a wearable nanosensor [220] for real-time monitoring of plant growth. This sensor adheres closely to the surface of leaves and accurately measures physiological parameters. Moreover, nanomaterials can be used in the real-time monitoring of plant cell cultures, for example, via fluorescent tracking technology to observe cellular growth and metabolism. Plant nanosensor technology—such as multi-walled carbon nanotube/copper flexible patches—has been adapted to wearable devices for infants and young children to track growth-hormone-related metabolites [221].

### 3.3. Application of Nanomaterials in Wearable Devices

Nanomaterials, owing to their unique physicochemical characteristics—such as large surface area, excellent electrical conductivity, tunable bandgap, favorable biocompatibility, and mechanical properties including stretchability and flexibility—are driving transformative advances in wearable sensing technologies. These materials, serving as either functional elements or structural components, significantly enhance the performance, integration, and multifunctionality of next-generation wearable platforms. This section reviews recent progress in nanomaterials-enabled wearable systems, categorized into electrophysiological sensors, electrochemical sensors, and multimodal integrated sensors.

#### 3.3.1. Electrophysiological Sensors

Wearable devices for electrophysiological monitoring aim to non-invasively capture signals such as ECG, EEG, EMG, and EOG, which require conformal interfaces with the skin and stable electrical contact.

**Figure 10:**
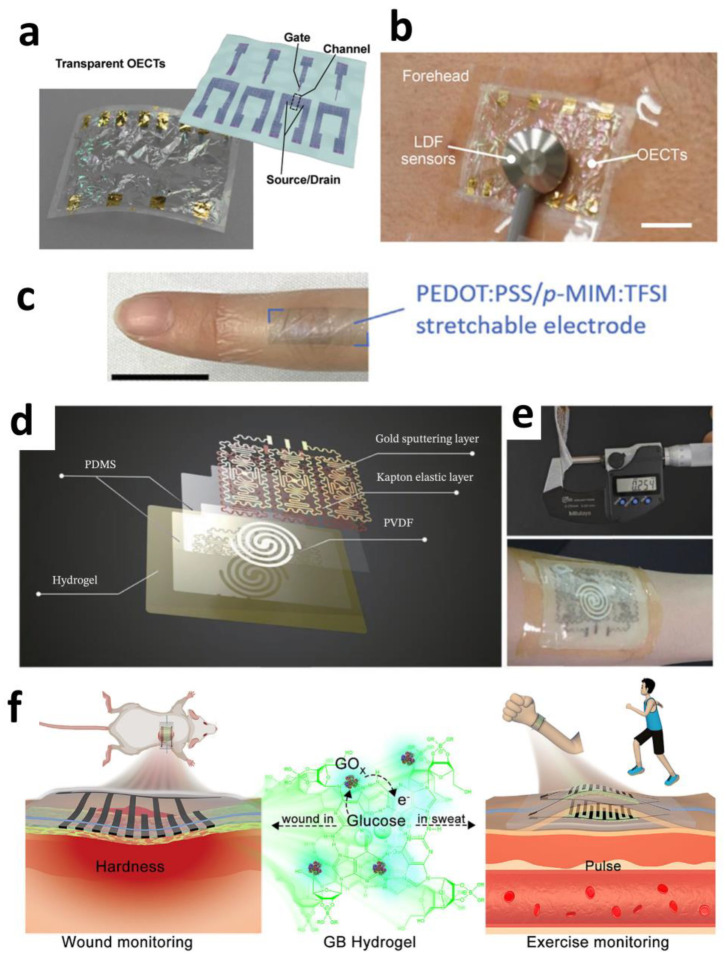
(**a**) Fully-transparent ultra-thin OECTs. (**b**) Photograph of LDF sensing directly above the OECTs attached on the forehead. The scale bar corresponds to 1 cm [222]. Copyright © 2022, the author(s). (**c**) Photograph of a stretchable and transparent PEDOT:PSS/p-MIM:TFSI/SEBS electrode attached to a forefinger. Scale bars: 2 cm [223]. Copyright © 2023, the author(s). (**d**) Explosion diagram of PCSP. (**e**) Display of photos of PCSP thickness measurement and attachment on skin [225]. Copyright © 2024, the author(s). (**f**) The diagram of functionalized LIG-mLPG for monitoring sweat glucose and physical quantities in animal and human models [228]. Copyright © 2025, the author(s).

Takemoto et al. manufactured a transparent electrode that can be used to monitor EEG and blood flow changes (Figure 10a,b). The fully transparent ultra-thin organic electrochemical transistor (OECT) platform leverages solution-processed Ag nanowire (AgNW) networks as source/drain electrodes and a ~180 nm PEDOT:PSS channel, all encapsulated between 1 µm parylene layers to yield >90% optical transparency. Electrode patterns are defined by selective wetting—hydrophilic parylene regions guide rod-coating of the AgNW dispersion to form ~25 Ω sq^−1^ networks—followed by thermal lamination at 5 MPa and 150 °C to seal the device and expose only the active channel. The resulting 2 µm thick OECT conformally adheres to skin and has been demonstrated as a wearable forehead sensor, simultaneously capturing EEG and optical blood-flow changes via laser Doppler flowmetry, as well as potentiometric nitrate-ion sensing for real-time stress biomarker monitoring [222].

In another study, Kim et al. employed a nanocomposite of PEDOT:PSS and the protic ionic liquid p-MIM:TFSI, solution-blended and spin-cast onto UV–ozone-treated SEBS elastomer to form a ~100 nm transparent (≥95% T), highly conductive (>450 S cm^−1^), and stretchable (>50% εc) electrode (Figure 10c); interconnects are made by Ag-paste bonding of copper wires, all annealed at 70 °C to stabilize the film. This skin-conformable patch, when adhered to fingers, face, or arm and connected to a compact instrumentation amplifier and tailored filters, reliably captures ECG PQRST waves, multi-directional EOG movements, and EMG muscle signals—maintaining low noise and mechanical resilience under bending and 3000 fatigue cycles—demonstrating its versatility for unobtrusive, multimodal health monitoring on exposed skin [223].

A stretchable dry electrode was also realized using Ag/AgCl NWs embedded in PDMS, fabricated via drop-casting of Ag NWs followed by electrochemical AgCl deposition (Wang et al.). The nanomaterial design leveraged Ag NWs for high conductivity and flexibility, while AgCl particles minimized polarization and enhanced signal stability. The resulting electrode exhibited exceptional stretchability (50% strain tolerance) and adhesion to curved skin surfaces without conductive gel. As a wearable patch, it achieved superior SNRs in multimodal biosensing: ECG (28.3 dB), EMG (28.9 dB), and EEG (6.6 dB), outperforming commercial wet electrodes. Applications included clinical-grade cardiac monitoring (validated via HRV analysis), muscle activity detection, and neural α-wave tracking, demonstrating its potential for comfortable, long-term health monitoring [224].

Furthermore, Xue et al. introduced a 250 μm thick patterned sensing patch (PCSP) integrating gold serpentine electrodes for sEMG and silver-plated PVDF fingerprint sensors for FMG (Figure 10d,e). The Au electrodes ensured low-impedance myoelectric signal acquisition, while the piezoelectric PVDF captured multidirectional muscle deformations. A thermo-responsive PVA/b-PEI/CaCl_2_ hydrogel enhanced skin adhesion, stabilizing signals during movement. The PCSP enabled synchronized mechanical–electrical monitoring of muscle activity, quantifying strength via grip tests (5–40 kg) and fatigue during repetitive curls. It further diagnosed Parkinson’s-related bradykinesia by detecting abnormal sEMG bursts and suppressed FMG outputs in ankle extensions. This device offers a compact solution for sports science and neuromuscular rehabilitation [225].

#### 3.3.2. Electrochemical Biosensors

Electrochemical wearable sensors detect biochemical markers in body fluids such as sweat, tears, and urine. Functionalized nanomaterials are essential in achieving high sensitivity, specificity, and resistance to biofouling.

Song et al. designed an antifouling, antimicrobial hydrogel composite (ABSACG), which is formed by drop-casting 3 μL of an amyloid albumin fibril (ABSA) precursor solution doped with Ti_3_C_2_T_x_ MXene nanosheets and CeO_2_ nanorods onto the working area of a flexible screen-printed electrode and incubating at 30 °C to gel. This creates a uniform, porous ABSACG coating over the carbon ink that transforms the SPE into a skin-conformable wearable patch. Leveraging the hydrogel’s hydration layer to resist fouling, the MXene to enhance electron transfer, and CeO_2_ for antimicrobial activity, the device delivers robust DA sensing from 0.05 to 300 μM (LOD 17 nM), retains over 90% sensitivity after 48 h in undiluted sweat, and maintains stable amperometric response through repeated bending, with real-sweat measurements matching ELISA within 7% RSD [226].

Another electrochemical system incorporated a silver nano dual-structure composite hydrogel (Ag NWs-Ag NCs/CCNF/Gel/PVA) for SERS-based detection of urinary biomarkers (Liu et al.). The Ag NWs and Ag NCs synergistically enhanced SERS sensitivity by generating electromagnetic “hot spots” at nanowire intersections and nanocube edges, achieving ultralow detection limits for urinary biomarkers (e.g., 69 nM for uric acid). The hydrogel was fabricated via freeze-thaw cycling of Gel/PVA embedded with Ag nanostructures, providing a porous matrix for urine absorption and analyte enrichment. Functionalization with 4-MBA enabled pH sensing through carboxylate state transitions. The sensor patches were adhered to diapers, forming a multiplexed platform that detected creatinine, uric acid, bilirubin, and pH in real urine with high accuracy (recovery: 88–107%) and antimicrobial properties (>99% bacterial inhibition). This system simplifies urine collection for vulnerable populations, leveraging hydrogel encapsulation to maintain sample freshness and SERS for rapid, non-invasive health monitoring [227].

#### 3.3.3. Multimodal and Multifunctional Wearable Sensors

Multimodal sensors leverage the integration of multiple signal types—electrical, biochemical, mechanical, or environmental—into a single wearable platform, often exploiting the multifunctionality of nanomaterials.

Li et al. pioneered a hybrid optoelectronic wearable system utilized LIG on PDMS to achieve dual-mode sensing. LIG—synthesized in situ via laser pyrolysis of PDMS—serves dual roles: as interdigital electrodes for electrochemical glucose sensing and as a refractive index modulator to create a microfiber long-period grating (mLPG) for biomechanical sensing. To ensure strain-insensitive glucose detection, LIG electrodes are functionalized with a guanosine-boric acid (GB) conductive hydrogel loaded with glucose oxidase (GOx), which provides enzymatic specificity and maintains conductivity under deformation. The resulting wearable device integrates optical mLPG and electrical (LIG/hydrogel) mechanisms into a single, compact patch, enabling simultaneous yet independent monitoring of biomechanical (e.g., pressure, arterial pulse) and biochemical (glucose) parameters. Applications include real-time tracking of diabetic wound healing in rats (hardness and glucose) and human exercise physiology (pulse and sweat glucose), demonstrating robust performance in complex biological environments (Figure 10f). This work advances wearable diagnostics by merging heterogeneous sensing modalities via nanomaterial engineering [228].

Moreover, a magnetic-field-coupled sensor (MFCS) was fabricated using a tilted PDMS dielectric embedded with NdFeB micropowders and screen-printed Ag/PET electrodes (Song et al.). Silver paste on PET ensures low-resistance conductors, while Nd_2_Fe_14_B powders embedded in PDMS provide rapid magnetic responsiveness. Assembled face-to-face and edge-sealed, this 3 mm thick patch conforms to skin or garments, enabling simultaneous pressure sensing (0.146 kPa^−1^, 12 ms response), proximity detection (−0.039 cm^−1^ at <2 cm), and magnetic field mapping (−1.72 T^−1^ at 60–230 mT) with clear signal discrimination. When worn, it captures vocal fold vibrations, tracks joint angles, monitors respiration and gait pressure, and deciphers contactless gestures—demonstrating a low-cost, scalable platform for next-generation wearable HMI and health-monitoring systems [229].

Additionally, Guo et al. developed a wearable multimodal perception system (MPA) using nanomaterial-functionalized fibers for integrated pressure, humidity, and temperature sensing. CNTs patterned on cotton enabled a 12-pixel pressure array with high sensitivity (6.45 kPa^−1^) and rapid response (30 ms), while TiO_2_-coated electrospun nanofibers achieved ultrafast humidity detection (1.4 s response). LiCl ions embedded in PVA/glycerol/cotton facilitated precise temperature resolution (2 °C). The entirely fiber-based design ensured exceptional breathability, validated by vapor transmission tests. The MPA was integrated with a microcontroller and Bluetooth for real-time environmental monitoring on the human palm, displaying pressure distribution, temperature, and humidity data on a smartphone. Additionally, the pressure array functioned as a flexible keyboard for a dual-password lock system, enabling secure wireless unlocking of electronic and physical interfaces. This work advances smart electronic skins for healthcare, robotics, and secure IoT applications [230].

Another multimodal sensor that is capable of monitoring physiological signals and temperature was designed by Yang et al. They fabricated stretchable transparent electrodes (STEs) using silver-nanoparticle-based serpentine grids embedded in a PEDOT:PSS/PDMS hybrid structure. The Ag grids provided high conductivity (29.9 Ω sq^−1^), while the PEDOT:PSS enhanced electrical connectivity and environmental stability. The PDMS substrate enabled exceptional stretchability (100% strain) and skin-like conformability. These STEs functioned as wearable multimodal sensors, wirelessly monitoring physiological signals (EMG, ECG) and temperature with high sensitivity. Additionally, they served as flexible touch panels for human–machine interfaces, maintaining performance under mechanical deformation. The work advances intelligent wearable electronics for healthcare and interactive systems [231].

Lastly, Pradhan et al. report the design and fabrication of a highly breathable, ultra-sensitive piezoresistive strain sensor based on two principal nanomaterial components: laser-engraved graphene (LEG) and a core–shell, hybrid metal–organic-framework-derived porous carbon (HPC). The LEG is produced by CO_2_ laser patterning of a polyimide film to yield a highly porous graphitic network with excellent cyclic stability but prone to microcrack-induced conductivity loss under large strains. To overcome this, the authors synthesize HPC via solvothermal growth of core–shell ZIF-8@ZIF-67 crystals followed by high-temperature carbonization. The resulting HPC nanoparticles, when deposited onto the LEG surface, slip into crack sites during stretching and re-establish conductive pathways, thereby dramatically increasing strain sensitivity. The synergy between LEG and HPC is evidenced by a reduction in the Raman D-to-G band ratio (ID/IG from 1.38 to 0.96) and an increase in BET surface area (from 2.18 to 3.06 m^2^ g^−1^) compared to bare LEG.

Electromechanically, the optimized LEG/HPC/PSEBS sensor achieves a gauge factor up to 575,542 in the 16–20% strain regime, with a limit of detection as low as 0.04%, and maintains a stable response over 9000 stretch–release cycles. When affixed to various body locations, the device reliably captures full-range joint motions (finger, elbow, knee), subtle arterial pulses—including jugular venous and wrist pulse waveforms with distinct A, C, X, V, Y and P, T, D peaks—and even speech and audio vibrations. These demonstrations underscore the multifunctionality of the nanomaterial-enabled wearable patch for real-time physiological monitoring, human–machine interfacing, and potential applications in speech rehabilitation and interactive electronics [232].

## 4. Summary and Outlook

This study systematically integrates the structure–property relationships of functional nanomaterials with the interfacial regulation mechanisms of bioelectrodes, thereby establishing a multi-scale correlation network spanning from intrinsic material characteristics to terminal application efficacy. A “material property-structural design-biomedical application” design framework is proposed here. This framework not only enables holistic parsing of the performance optimization pathways for bioelectrodes but also, by revealing the mapping rules between nanoscale regulation and macroscopic biomedical functions, provides a universal theoretical framework for the rational design of high-performance bioelectrodes. Concurrently, through systematic analysis of typical cases, it clarifies the key bottlenecks and breakthrough pathways in translating laboratory prototypes to clinical applications, thereby establishing a complete system for the development of intelligent bioelectrodes that bridges fundamental innovation to technological implementation.

Dynamic bio-interface adaptation is emerging as a central proposition for long-term implantation. During prolonged implantation, the biological responses to implants evolve over time, yet static interfaces lack adaptive mechanisms to address these temporal changes. Consequently, there is an urgent need to develop environmentally responsive smart materials whose surface physicochemical properties can undergo real-time modulation in response to biochemical signal variations in the local microenvironment, thereby achieving self-balancing between antifouling properties and electrochemical activity [233]. Multimodal sensing integration requires deeper system-level convergence: next-generation platforms must unify cross-scale physiological signal acquisition, incorporating bioinspired fluidic control and on-chip photonics to construct spatiotemporal-resolved networks. Artificial-intelligence-driven edge computing will enable real-time correlation mining of heterogeneous data, generating dynamic bioprofiles. Clinical translation necessitates manufacturing paradigm shifts—medical imaging-guided additive manufacturing should advance patient-specific device customization, facilitating the deployment of “monitoring-diagnosis-intervention” closed-loop systems (e.g., intelligent therapeutic devices triggered by neural signature recognition). The transformation of energy supply modes is particularly critical. Conventional power solutions, including energy storage devices (batteries/capacitors) and wireless power supply systems (metal coils), generally suffer from volume constraints and biotoxicity risks (e.g., electrolyte leakage and heavy metal ion release). Self-powered designs leveraging endogenous bioenergy, such as novel endogenous power supply solutions including biofuel cells [234] and triboelectric nanogenerators [235], can effectively mitigate these issues.

Critically, ethical and industrial framework construction has become a prerequisite for technological deployment. Globally accessible biocompatibility databases must catalog material trafficking routes and metabolic fates in complex physiological milieus. Neuromorphic encryption-based security architectures should safeguard against data breaches and unauthorized manipulation. Transdisciplinary medical–engineering collaboration mechanisms, coupled with regulatory science research, will accelerate clinical pathways.

As environmentally responsive materials, flexible electronics, and neuromorphic computing deeply converge, bioelectrodes will evolve from single-function tools into integrated living systems endowed with environmental perception, autonomous decision-making, and adaptive modulation. This transformative paradigm shift will not only redefine therapeutic strategies for critical diseases but also catalyze proactive health ecosystems centered on “predictive intervention,” ultimately culminating in a fundamental transition from disease treatment to sustainable health preservation.

## Figures and Tables

**Table 1:** Timeline of the development of electrophysiological/electrochemical electrode technology.

Development Period	Development Situation	References
1920s–1950s	Early metal and glass microelectrodes	[24,25]
1950s–1960s	Standard reference electrode and enzyme electrode	[26,27]
1970s–1980s	Silicon needle array microfabrication and arrayed	[26,28]
1990s	Flexible polyimide substrate	[29]
2000s	Interface modification of conductive polymers	[30,31]
2010s	Nano-carbon and ultra-high-density probes	[32,33]
2020s	Two-dimensional nanomaterials and bio-biomimetic hydrogels	[34,35]
This review		
2020–2025	The performance improvement brought by functional nanomaterials for bioelectrode interfaces, long-term stability and anti-biofouling technology integration	-
2021–2025	Multimodal integration and closed-loop neural regulation	-
2023–2025	Microstructure design promotes performance improvement	-

## Data Availability

Not applicable.

## Abbreviations

The following abbreviations are used in this manuscript:

ALP	Alkaline Phosphatase
AMMP	Aerosol Multi-Material Printing
BIEF	Built-In Electric Field
BMIs	Brain–Machine Interfaces
CEA	Carcinoembryonic Antigen
CGM	Continuous Glucose Monitoring
CIC	Charge Injection Capacity
CIL	Charge Injection Limit
CNTs	Carbon Nanotubes
COFs	Covalent Organic Frameworks
Cor	Cortisol
CV	Cyclic Voltammetry
DA	Dopamine
DBS	Deep Brain Stimulation
DPV	Differential Pulse Voltammetry
EC	Electrochemical
ECG	Electrocardiography
ECL	Electrochemiluminescence
ECoG	Electrocorticography
EEG	Electroencephalographic
EMG	Electromyographic
EOG	Electrooculography
ER	Endoplasmic Reticulum
eTPU	Electrospun Thermoplastic Polyurethane
GB	Guanosine-Boric Acid
GCE	Glassy Carbon Electrode
GFP	Green Fluorescent Protein
Glu	Glutamate
GPNMB	Glycoprotein Non-Metastatic Melanoma Protein B
IL	Ionic Liquid
ISF	Interstitial Fluid
ISFET	Ion-Sensitive Field-Effect Transistor
LFP	Local Field Potential
LIG	Laser-Induced Graphene
LM	Liquid Metal
LOD	Limit Of Detection
LSPR	Local Surface Plasmon Resonance
MFCS	Magnetic Field-Coupled Sensor
MIECs	Mixed Ionic-Electronic Conductors
mLPG	Microfiber Long-Period Grating
MOFs	Metal-Organic Frameworks
MONPs	Metal Oxide Nanoparticles
MOVPE	Metal-Organic Vapor Phase Epitaxy
MUA	Multi-Unit Activity
MWCNTs	Multi-Walled Carbon Nanotubes
NCS	Non-Convulsive Seizures
ND	Nanodiamond
NPs	Nanoparticles
NT-3DFG	Nanowire-Templated Three-Dimensional Fuzzy Graphene
NWs	Nanowires
OECT	Organic Electrochemical Transistor
PAA	Poly(Acrylic Acid)
PD	Parkinson’S Disease
PDA	Polydopamine
PEDOT:PSS	Poly(3,4-Ethylenedioxythiophene)-Poly(Styrenesulfonate)
PEGDA	Poly(Ethylene Glycol) Diacrylate
POC	Point-Of-Care
PU	Polyurethane
PVA	Polyvinyl Alcohol
QDs	Quantum Dots
SEAs	Surface Electrode Arrays
SMR	Signal-To-Motion Artifact Ratios
SNMs	Silica Nanoporous Membranes
SNR	Signal-To-Noise Ratio
SPR	Surface Plasmon Resonance
ST	Serotonin
STEs	Stretchable Transparent Electrodes
SWV	Square-Wave Voltammetry
TBI	Traumatic Brain Injury
TEM	Transmission Electron Microscope
